# Advancing the 3Rs in bone tissue engineering: emerging *in vitro*, *in silico*, and refined *in vivo* strategies

**DOI:** 10.3389/fphys.2026.1811508

**Published:** 2026-05-11

**Authors:** Adelaide L. Cagle, Afton K. Limberg, Amritha Anup, Aleyna E. La Croix, Katherine R. Hixon

**Affiliations:** 1 Thayer School of Engineering, Dartmouth College, Hanover, NH, United States; 2 Geisel School of Medicine, Dartmouth College, Hanover, NH, United States

**Keywords:** 3Rs principles, animal models, bioprinting, bone tissue engineering, *in silico* modeling, *in vitro* modeling, organoids, translational research

## Abstract

Tissue engineering is an interdisciplinary field integrating materials science, biology, chemistry, and immunology to develop regenerative solutions for complex tissues. Validation of tissue-engineered therapies, however, remains challenging—particularly for bone, whose hierarchical organization governs vascularization, mechanical loading, and continuous remodeling. Historically, these complexities have necessitated extensive reliance on *in vivo* animal models. Here we examine contemporary advances in modeling and validating bone tissue engineering strategies through the framework of the 3Rs principles: Replacement, Reduction, and Refinement. This review summarizes recent progress in advanced *in vitro* platforms, including organoids, bioprinting, and organ-on-a-chip systems, as well as *in silico* modeling approaches designed to decrease reliance on animal experimentation. We also highlight evolving best practices in experimental design, longitudinal imaging, welfare optimization, and reporting standards that improve the ethical and scientific conduct of necessary animal studies. Drawing primarily from literature published since 2020, this review provides an updated assessment of how the 3Rs are being operationalized within bone tissue engineering. Although significant technical and biological limitations remain, particularly in replicating vascularized, mechanically competent, and immunologically integrated bone tissue, the field demonstrates measurable progress toward Reducing animal use and improving translational relevance. Collectively, current developments reflect a realistic yet optimistic trajectory in which non-animal methodologies increasingly Replace *in vivo* work while also highlighting achievable short-term strategies to Refine necessary animal research.

## 1 Introduction

The principles of 3Rs were introduced by Russell and Burch in 1959 to encourage the scientific community to recognize and minimize the ethical costs of animal experimentation. Since their inception, the 3Rs have become foundational to the ethical governance of animal research and are now embedded in national and international legislation, funding policies, and institutional review processes (Grimm et al., 2023). Russell and Burch originally defined the 3Rs as follows (Scribd):

Replacement: the substitution of conscious living higher animals with non-sentient material;

Reduction: the use of fewer animals to obtain information of a given amount and precision;

Refinement: any modification that decreases the severity or inhumanity of procedures applied to animals that must still be used.

Since their conception, both the interpretation and implementation of the 3Rs have evolved alongside advances in science and changes in societal and regulatory perspectives. In 2024, the New York Declaration on Animal Consciousness broadened discussion of sentience to include a wider range of vertebrate, and in some contexts invertebrate, species (The New York Declaration on Animal Consciousness). This expansion has implications for how Replacement is conceptualized and operationalized. In parallel, technological progress in tissue engineering, microphysiological systems, and computational modeling has created new opportunities to implement the 3Rs in ways that were not envisioned at the time of their introduction.

Despite progress, the scale of animal studies in research remains extensive. In the United States, use of 775,297 animals was reported in 2024, an increase from 774,065 in 2023, with the majority classified as undergoing procedures involving no or minimal pain (Research Facility Annual Report Summary, 2024). The animals included in this report were dogs, cats, hamsters, rabbits, guinea pigs, nonhuman primates, sheep, pigs, birds, other farm animals, and other mammals (Research Facility Annual Report Summary, 2024). Notably, these figures exclude rats and mice, which constitute the majority of mammals used in biomedical research and are also the primary species used in musculoskeletal and bone tissue engineering studies (Why animal experiments are still indispensable in bone research: a statement by the european calcified tissue society, 2023). As a result, the true extent of animal use is difficult to quantify. A widely cited estimate made by Carbone in 2021 suggested that approximately 111 million rats and mice were used in United States research in 2017 (Carbone, 2021), highlighting a significant gap between reported statistics and actual practice. However, detailed statistics specific to musculoskeletal research remain unavailable, limiting the ability to precisely quantify animal use within this field. Additionally, limited reporting on procedure severity and distress as well as favorable alternatives complicates evaluation of progress toward the core ethical aim of the 3Rs: minimizing animal suffering (Tannenbaum and Bennett, 2015; Critser and Locke, 2024). Together, these discrepancies highlight both the scale of animal use and the limitations of current reporting frameworks for assessing progress toward the 3Rs. These numbers underscore the continued reliance on animal models in biomedical research and emphasize the importance of advancing strategies that support the ethical principles of the 3Rs.

However, developing alternatives remains challenging in fields where complex tissue structure, mechanical loading, and multicellular interactions must be faithfully recapitulated. This challenge is particularly pronounced in musculoskeletal systems, where structure-function relationships are tightly coupled across multiple spatial and temporal scales. Musculoskeletal research presents a particular challenge due to the structural, mechanical, and multicellular complexity of bone and its interface with surrounding tissue, which have traditionally necessitated extensive animal experimentation. Bone tissue engineering represents a particularly high-impact and high-burden area of preclinical research (Ghanem et al; Huang et al., 2022; Flores et al., 2024). Bone defects arising from trauma, tumor resection, congenital anomalies, infection, and degenerative disease constitute a major clinical challenge worldwide, with substantial personal and socioeconomic costs (Ghanem et al; Huang et al., 2022; Flores et al., 2024). As the second most transplanted tissue after blood, bone has long been a focus of regenerative medicine efforts (Gillman and Jayasuriya, 2021). However, the hierarchical structure of bone, spanning mineralized matrix, vascular networks, immune components, and mechanically responsive cell populations, has historically limited the predictive power of simplified *in vitro* systems and reinforced reliance on animal models for validation (3D bioprinting bone/cartilage organoids: construction, applications, and challenges; Stein et al., 2023; Rodriguez-Palomo et al., 2024; Hadjiargyrou et al., 2026). This reliance is further compounded by the need to assess load-bearing function, long-term remodeling, and host integration, outcomes that are difficult to capture without *in vivo* studies (Stein et al., 2023; Hadjiargyrou et al., 2026). For these reasons, bone tissue engineering serves as a stringent test case for the practical implementation of the 3Rs. Advances that enable meaningful Replacement, Reduction, or Refinement in this context have the potential to produce outsized ethical and scientific benefits, not only within musculoskeletal research but across the broader landscape of preclinical biomedical science. The emergence of sophisticated co-culture systems, organ-on-a-chip (OoC) technologies, and *in silico* modeling offers a path toward recapitulating human physiology more accurately than traditional animal models.

In a significant shift toward the adoption of the 3Rs in scientific research, the United States Food and Drug Administration (FDA) has recently moved away from requiring animal studies as a default prerequisite for investigational new drug (IND) applications, emphasizing the development and use of non-animal standards and complementary advanced *in vitro* and *in silico* strategies (US - Research). By establishing credible non-animal approaches as default options for early-stage evaluation, the field can meaningfully Reduce reliance on animal experimentation while improving translational relevance. This review examines current and emerging strategies aligned with the 3Rs in bone tissue engineering, highlighting their potential to Reduce or eliminate reliance on animal models while strengthening prediction of human outcomes and translational relevance. The review is organized to first evaluate limitations of current animal models, followed by emerging Replacement strategies including *in vitro* and *in silico* systems, and finally approaches to Reduction and Refinement that improve both ethical and scientific outcomes in bone tissue engineering.

## 2 Data and methods

### 2.1 Data sources

Data were gathered from PubMed and Google Scholar searches, and references from relevant studies. Keywords used in the search were: ‘3Rs’, ‘animal replacement’, ‘animal reduction’, ‘animal refinement’, ‘animal model’, ‘tissue engineering’, ‘bone’, ‘musculoskeletal’, ‘animal guidelines’, ‘bone organoid’, ‘bone-on-a-chip’, ‘2D bone culture’, ‘3D bone culture’.

### 2.2 Inclusion and exclusion criteria

Inclusion criteria included literature directly related to one of the elements of the 3Rs that could be applied in, or related directly to, the bone tissue engineering space. This review focused primarily on literature that discussed best practices or discrepancies in animal models used for bone tissue engineering as well as the advantages, drawbacks, and recent developments in animal model alternatives.

Exclusion criteria suspended literature not relevant to advancements or shortcomings in *in vivo* bone tissue research, publications that did not address at least one of the 3Rs principles, and models unrelated to simulating bone or its immediate supporting tissues. The review primarily focused on literature published within the past five years (post-2020), with the exception of specific seminal papers included where appropriate.

## 3 Traditional models and limitations

Historically, bone tissue engineering has relied on a sequence of models to screen materials, study interactions among cells, biologics, and/or implants, and engineer repair processes to determine benefits and risks. Classical *in vitro* cultures are used to investigate cell differentiation, fundamental biological pathways, and drug responses. When early-stage findings warrant further investigation, animal models are typically employed to examine translational aspects of the research.

**Figure 1:**
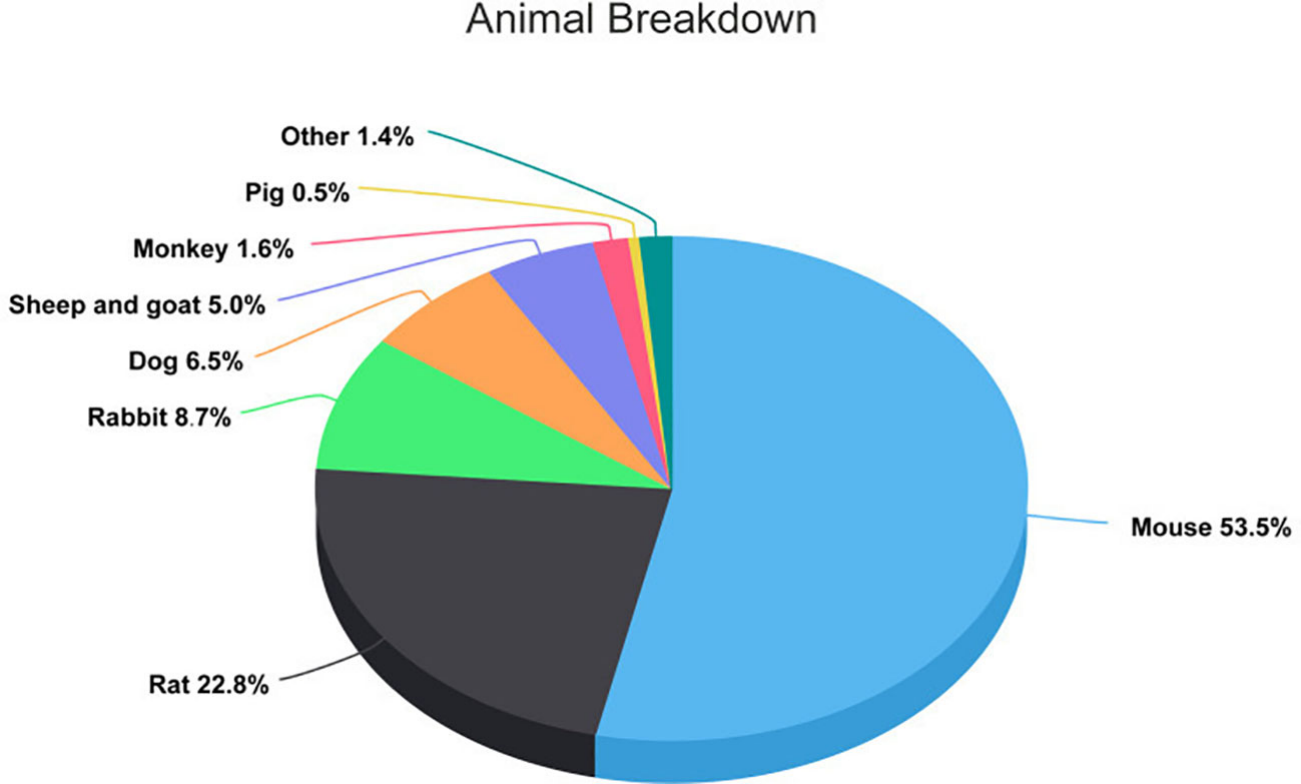
Distribution of animal models used in preclinical fracture studies from 2013-2023. Reproduced from Gao et al., 2023 (Bioengineering), licensed under CC BY 4.0 (https://creativecommons.org/licenses/by/4.0/deed.en).

Animal models remain a gold standard for early-stage investigations of musculoskeletal conditions, potential drug targets, and therapies, playing a central role in translating discoveries from the bench to the bedside. Small animal models (i.e., mice and rats) are often the first step due to their size, ease of housing, short reproductive cycles, well-characterized biology, and extensive genetic toolkits. These include genetically modified strains and disease models (e.g., ovariectomized rats as a model for postmenopausal bone loss in women; genetically modified mice for interrogating signaling pathways critical in osteoporosis) (Hixon and Miller, 2022; Koh et al., 2024). These features make rodent models highly effective for proof-of-concept and discovery studies. However, important caveats exist; for example, rodent growth plates do not fully close, which can complicate studies of aging-associated bone loss. Even so, mice and rats comprise the majority of animals featured in fracture injury and healing models (**Figure 1**) (Gao et al., 2023).

**Figure 2:**
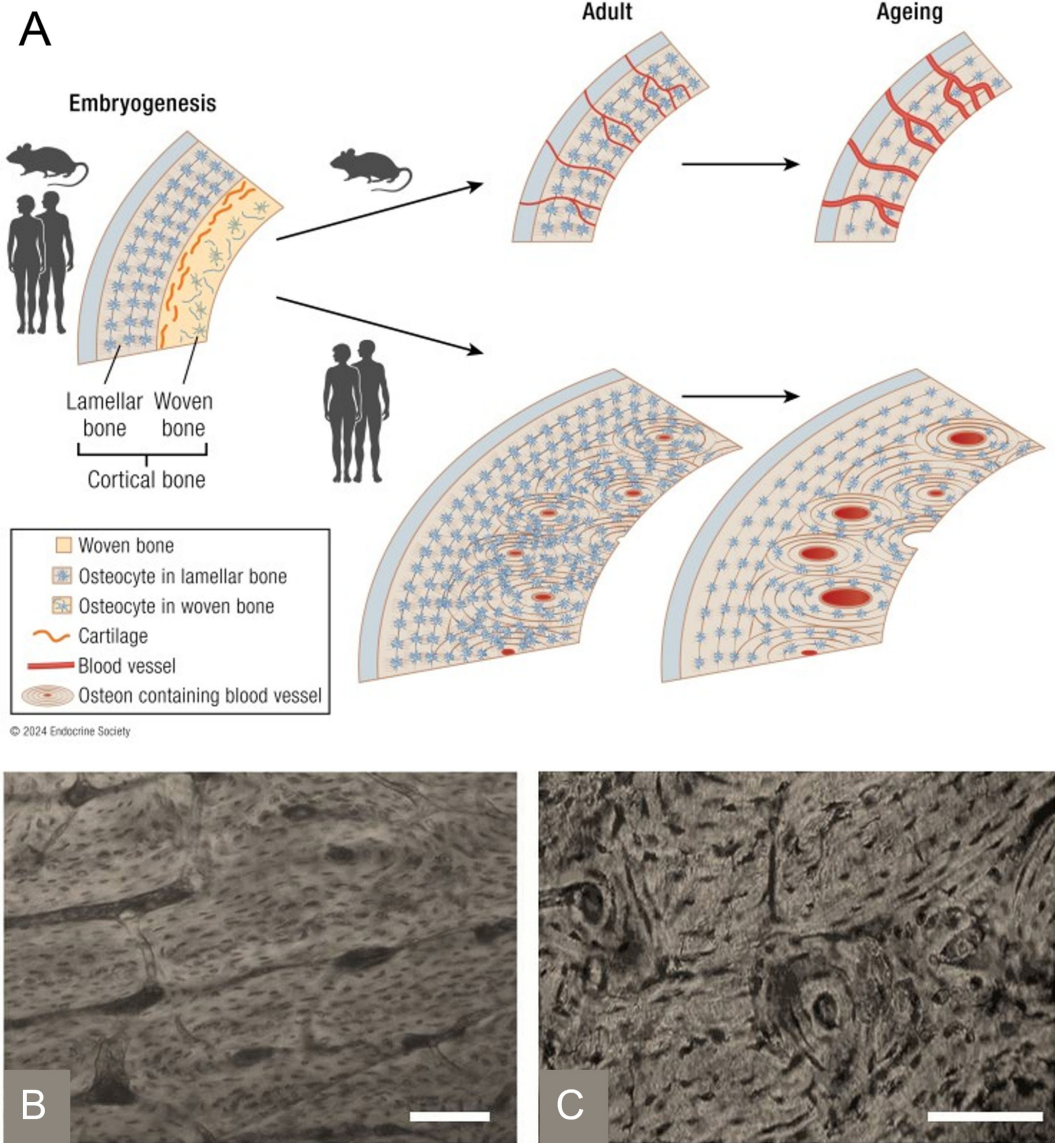
Anatomical organization of the Haversian system within osteons in large animal models. **(A)** Schematic diagram illustrating the hierarchical organization of Haversian canals and osteons within mature cortical bone in humans showing the reduced organization present in most rodent bones compared to the highly organized Haversian systems in humans. Reproduced from Koh et al., 2024 under the Creative Commons Attribution License (CC BY 4.0 https://creativecommons.org/licenses/by/4.0/deed.en). **(B)** Longitudinal histological section of bovine humerus with scale bar = 80 µm and **(C)** transverse histological section of bovine humerus with scale bar = 80 µm. Reproduced from Zedda M. and Babosova R., 2021 (Zoomorphology), licensed under CC BY 4.0 (https://creativecommons.org/licenses/by/4.0/deed.en).

Rodent models have nonetheless enabled foundational discoveries in skeletal biology. Reverse genetics in rodents contributed to identification of systemic regulators such as the RANKL/osteoprotegrin axis on osteoclast formation and bone physiology (Li et al., 2000; Stein et al., 2023), and clarified sclerostin’s role in bone homeostasis through regulation of canonical Wnt signaling, helping motivate therapeutic development (e.g., anti-sclerostin antibody for the treatment of osteoporosis) (Li et al., 2000; Winkler et al., 2003; Tanaka and Matsumoto, 2021; Stein et al., 2023). However, due to their size, rats and mice exhibit limited intracortical remodeling and poorly developed Haversian systems. Full Haversian canals are vital for nutrient transfer and organization within large bone systems (Zedda and Babosova, 2021). In rodents, Haversian-like structures, while present, are small and typically confined to regions of thicker cortical bone (**Figure 2**), rather than being broadly distributed as in human cortical bone. These structural differences limit the ability of rodent models to accurately capture cortical remodeling and long-term bone adaptation observed in human systems (Koh et al., 2024).

Rabbits, in contrast, possess Haversian remodeling which allows for closer approximation of human cortical bone structure and mechanical behavior compared to rodent models (Koh et al., 2024). This feature makes rabbits particularly valuable for studies in which cortical architecture, osteon remodeling, and load-bearing mechanics are central to the research question. Rabbits, such as the New Zealand White Rabbit, are widely used in orthopedic research and reach skeletal maturity at approximately 20–30 weeks of age (Schafrum Macedo et al., 2019; van Agtmaal et al., 2024). Their size and cortical structure make them well-suited for implant-based investigations, particularly studies evaluating fixation strategies and biomaterial-mediated bone regeneration. For example, recent work has utilized rabbit critical-sized defect (CSD) models in both the cranium (non-load bearing) and ulna (load bearing) to investigate repair using synthetic bone substitutes. In this study, porous calcium phosphate ceramics, including porous beta-tricalcium phosphate (β-TCP) and biphasic calcium phosphate, were implanted into rabbit CSDs to investigate the bone regeneration capability of these materials. The results demonstrated complete cranial defect repair at 12 weeks and ulna union by 24 weeks, highlighting the model’s utility for assessing biomaterial performance across different mechanical environments (Lei et al., 2025). Beyond segmental defect repair, femoral condyle rabbit models have been widely employed to investigate joint mechanics, as well as bone (Sadek et al., 2023; Alonso-Fernández et al., 2024) and osteochondral regeneration (Guo et al., 2020; Zhou et al., 2024). These models provide a platform for studying subchondral bone-cartilage interactions under physiologically-relevant loading conditions. Rabbits have also been adapted to model metabolic bone disease. An osteoporosis model induced by ovariectomy combined with glucocorticoid administration has been established and used to mimic postmenopausal and secondary osteoporosis (Baofeng et al., 2010; Sadat-Ali et al., 2021). Use of this model has elucidated the anabolic effects of parathyroid hormone (PTH) on osseointegration, particularly following dental implant placement in the femoral epiphyses (Oki et al., 2022). It has additionally supported investigations into osteoporosis-associated cortical bone porosity and structural degradation (Harrison et al., 2020).

Larger animal models can better approximate human-scale loading, bone size, and remodeling patterns and are often used for implants, fracture fixation, and joint/spine research. Sheep and goat models have body weights comparable to humans and long-bone dimensions well-suited for testing human sized implants. Sheep models (e.g., Dorset/Rideau Arcott rams) have been used to compare porous coatings including Gription and Porocoat in the femoral condyle region (Changoor et al., 2022), and ovine cadaver legs have supported studies of electric field penetration from noninvasive bone stimulator therapy (Verma et al., 2022). *In vivo* sheep osteotomy models, including non-critical and tibial CSDs stabilized with compression plates, have been used to test combined electric and magnetic field therapies (Darwiche et al., 2023). Additionally, the infraspinous scapular region of sheep has been utilized to assess a three dimensional (3D)-printed *in vivo* bioreactor platform placed at the bone-periosteum interface (Ghosh et al., 2024).

Dog models are widely used in dental and alveolar bone research, particularly in studies focused on restoring bone volume to support dental implant placement (Mosaddad et al., 2024). Their bone and mineral density, secondary osteon formation, intracortical remodeling, tooth anatomy, and wound healing similarity to human models make dog models valuable for translational implant investigations (Mosaddad et al., 2024). The osteoconductive bio-ceramic material β-TCP is widely studied for its capacity to support bone regeneration and osseointegration (Lu et al., 2021). Several studies have investigated the impact of β-TCP/collagen composites in alveolar defects in canine models (Ho et al., 2016; Hojo et al., 2020; Eldeeb et al., 2023; Yoshino et al., 2023; Kim et al., 2025), as well as multi-component strategies implementing a combination growth factors (i.e., platelet-derived growth factor), bone marrow-derived mesenchymal stem cells (BMSCs), β-TCP particles, and other mineral graft materials (Xu et al., 2016; Fukuba et al., 2021; Takeuchi et al., 2023). These studies highlight the role of canine models in evaluating combined biomaterial-biologic strategies under clinically relevant loading and healing conditions. Beyond dental applications, dog models have also been employed to investigate treatments for osteoporotic fractures affecting the hip or spine. One example is the use of a local osteo-enhancement procedure in which the triphasic, calcium-based osteoconductive material AGN1 was implanted in a proximal CSD in skeletally mature hounds (Shaul et al., 2022). This approach enabled assessment of structural reinforcement and bone regeneration in anatomically and mechanically relevant contexts.

Pig models are also favorable for bone studies due to similarities in bone anatomy, metabolism, healing capacity, remodeling pathways, and mineral density relative to humans (Li et al., 2015). Recent examples include the first porcine osteogenesis imperfecta model in 2026 (Aguirre-Flores et al., 2026), a mandibular osteomyelitis model assessing cell-microparticle combination therapy in 2019 (Hwang et al., 2019), and evaluation of nanocomposite fibrous scaffolds for mandible reconstruction in 2022 (Unnikrishnan et al., 2022).

Although these models have advanced scientific understanding of material-biology interactions, important limitations remain, as Haversian remodeling—characteristic of human cortical bone—is present in humans and several large animals but is limited or underdeveloped in rodents (Gao et al., 2023; Lad, 2023). Despite these limitations, rodent models remain useful for investigating conditions associated with increased cortical porosity (Koh et al., 2024). Another important consideration when using these models is the protective effects observed in young and growing rats and mice (Koh et al., 2024), which can mask the impact of disease states and therapeutic interventions. Even when anatomical similarity exists, model-specific constraints can be substantial; for example, pigs present challenges related to rapid growth, behavior, and handling (Li et al., 2015).

Short- to mid-term healing processes are well-captured in rabbit models due to their rapid vascular response; however, this accelerated healing limits their ability to predict long-term outcomes and remodeling dynamics relevant to human bone repair (Varut et al., 2025). In general, larger animals are preferred for load bearing applications (Varut et al., 2025). Additionally, most commonly used models are quadrupeds, which differ fundamentally from humans in gait, loading distribution, and posture. Non-human primates exhibit closer biomechanics (i.e., bipedalism) but are rarely used due to ethical constraints and are generally reserved for late-stage validation. For example, in 2022, xenografts from a pig knockout model were tested in rhesus monkeys with cylindrical femoral condyle defects (Wang et al., 2022), focusing on xenograft rejection and promotion of new bone formation.

Collectively, species-specific differences in bone structure, healing kinetics, and remodeling capacity highlight the limitations of traditional animal models in predicting human outcomes. Despite the many benefits of using animal models for studying bone healing in response to therapeutics and implants, these limitations and associated ethical considerations underscore the need to develop and adopt alternative *in vitro* and *in silico* systems that offer more controllable, scalable, and ethically responsible approaches to studying bone regeneration.

## 4 Replacement: *in vitro* and *in silico* systems

Replacement is the first pillar of the 3Rs framework and refers to strategies that avoid or substitute the use of live animals in research and prioritize testing with non-sentient systems. Replacement approaches can be broadly categorized as either: *full* or *partial* Replacement. Full Replacement eliminates animal use entirely through non-animal alternatives such as *in vitro* culture systems, microphysiological platforms, and computer-based or *in silico* models. Partial Replacement lessens reliance on live animals by utilizing methods in which animal models do not experience pain or distress, animal involvement is indirect, or species considered “non-sentient” are used (Animal Use Alternatives (3Rs)). As scientific understanding and definition of animal sentience continues to evolve, the impetus to develop fully non-animal systems has further strengthened (The New York Declaration on Animal Consciousness).

Since the introduction of the 3Rs principles, Replacement technologies have evolved substantially, progressing from simple two-dimensional (2D) monolayer cell cultures to increasingly complex and physiologically relevant systems. Importantly, effective *in vitro* Replacement does not require full replication of an entire native tissue or organ; rather it requires modeling the specific biological, mechanical, or biochemical features relevant to the research question (Caddeo et al., 2017). However, musculoskeletal (bone) biology involves multicellular interactions, mechanical signaling, and specialized interface interactions, necessitating higher levels of model complexity to address many clinically relevant questions.

Recent advances include co-culture systems, dynamic bioreactors, microfluidic OoC platforms, and 3D culture systems such as organoids and scaffold-based constructs. In parallel, computational and *in silico* models have emerged as powerful tools to simulate biological processes and guide experimental design. Collectively, these approaches expand the capacity to model musculoskeletal biology and pathology in human-relevant systems while Reducing or Replacing reliance on animal experimentation. Because bone tissue function depends on extensive cellular crosstalk and the formation of specialized tissue interfaces (e.g., bone-muscle and -tendon junctions), it is essential that *in vitro* models capture these multicellular and biomechanical interactions. **Table 1** summarizes representative *in vitro* and *in silico* systems used to support Replacement strategies in bone tissue engineering, along with their primary applications and limitations.

**Table 1:** Representative *in vitro* and *in silico* systems supporting Replacement strategies in bone tissue engineering.

Example system	Representative systems	Best use/replacement opportunities	Limitations (where animal models may still be needed)
2D culture systems			
2D Cell Culture	Monolayer cultures of osteoblasts, myoblasts, MSCs (Miki et al., 2012; Yuste et al., 2021; Allen et al., 2023; Lipreri et al., 2023; Yamada et al., 2023)	High-throughput drug screening, gene expression studies, mechanistic signaling pathways, toxicity testing	Limited representation of tissue architecture; lacks multicellular interactions and biomechanical cues
2D Co-culture Systems	Bone-muscle or bone-tumor co-cultures (Calejo et al., 2018; Yuste et al., 2021)	Studying paracrine signaling and intercellular communication between musculoskeletal tissues	Still lacks spatial organization and mechanical loading environment
Micropatterned/Topographical Substrates	Grooved substrates, stiffness-controlled hydrogels (Engler et al., 2006; Tay et al., 2011; Li and Kilian, 2015; Jenkins and Little, 2019; Ragni et al., 2025; Cojocaru et al., 2026; Hassan et al., 2026; Persson et al., 2026)	Investigating mechanotransduction, cell alignment, and lineage commitment	Limited to simplified geometries; does not capture full 3D tissue complexity
Bioreactor-Enhanced Culture	Perfusion or mechanically stimulated systems (Martin et al., 2004; Zhang, 2022,)	Investigating mechanobiology and tissue maturation under controlled dynamic stimulation	Increased complexity and cost; limited scalability and standardization
3D culture systems			
Organoids/Self-assembled tissues	Stem-cell derived bone, muscle, or neuromusculoskeletal organoids (Akiva et al., 2021; Liu et al., 2025; Zhang et al., 2025,)	Modeling development, disease mechanisms, and multicellular organization	Limited structural maturity; challenges with vascularization and long-term remodeling
Scaffold-based Constructs	Hydrogels, cryogels, electrospun scaffolds (Lee et al., 2022; Yang et al., 2023; Yin et al., 2025)	Modeling cell-matrix interactions, tissue regeneration, and engineered tissue interfaces	May not fully replicate native mechanical properties or biological complexity
Gradient Scaffolds	Composition or stiffness gradients mimicking entheses (Seidi et al., 2011; Marrella et al., 2016; D’Amora et al., 2018; D’Amora et al., 2018; Vesvoranan et al., 2022)	Studying interface biology (e.g., tendon-bone, cartilage-bone transitions)	Difficult to fully reproduce native gradients and long-term integration
Bioprinted Constructs	3D printed bone or osteochondral tissues (Anada et al., 2019; Zhang et al., 2020; Alcala-Orozco et al., 2021; Sun et al., 2022; Huang et al., 2025)	Modeling spatially organized tissues and vascularized constructs	Limited mechanical strength; challenges in material selection and scalability
Microphysiological systems			
Organ-on-a-Chip (OoC) Platforms	Bone-on-a-chip or muscle-bone systems (Ajalik et al., 2022; Leung et al., 2022,)	Modeling dynamic biochemical gradients, fluid flow, and multi-tissue interactions	Device variability, limited standardization, and challenges in long-term culture
Computational/*In silico* models			
Finite Element Models of healing	Predicting tissue differentiation, fracture healing, or implant mechanics (Barkaoui et al., 2016; Jung and Buehler, 2017; Fielder and Nair, 2024; Yang et al., 2024)	Predicting tissue differentiation, healing outcomes, and mechanical behavior	Requires experimental validation and accurate biological input parameters
Digital Twins/AI Models	Patient-specific musculoskeletal simulations (Handelman et al., 2018; Javaid et al., 2023; Wu et al., 2024; Fu et al., 2025)	Virtual testing of implants, treatment strategies, and personalized biomechanics	Limited by data availability, model interpretability, and validation constraints

### 4.1 2D *in vitro* culture systems

Overall, 2D cell culture systems remain the most widely used *in vitro* platforms in musculoskeletal research, particularly in bone, due to their simplicity, cost-effectiveness, and capacity for high-throughput experimentation (Duval et al., 2017; Jensen and Teng, 2020). Monolayer cultures enable rapid assessment of cellular responses to biochemical and mechanical stimuli, and can incorporate patient-derived *ex vivo* cells (Allen et al., 2023), supporting personalized investigation while avoiding animal use. These foundational 2D systems first established the feasibility of Replacing animal models for early-stage screening and mechanistic studies.

However, the physiological relevance of traditional monolayer cultures is limited by their inability to recapitulate multicellular organization, spatial heterogeneity, and inter-tissue communication central to musculoskeletal function (Miki et al., 2012; Yuste et al., 2021; Yamada et al., 2023). To address these limitations while preserving experimental accessibility, enhanced 2D platforms have been developed that incorporate interface-relevant cues such as paracrine signaling, spatial organization, geometry-controlled cellular behavior, and dynamic culture conditions (Yuste et al., 2021).

Collectively, indirect and direct 2D platforms enable interrogation of tissue-tissue crosstalk that is inaccessible in monoculture while preserving throughput and analytical simplicity. For example, an indirect 2D co-culture of C2C12 myoblasts and TE85 osteosarcoma cells demonstrated robust myotube formation alongside upregulation of osteogenic markers (including RUNX2/CBFa1 and osteocalcin) in the bone compartment, effects absent in monoculture. These findings underscore the critical role of intercellular communication even within simplified planar systems (Wragg et al., 2020).

Dynamic stimulation further enhances the physiological relevance of 2D culture platforms by introducing biochemical, mechanical, and environmental cues. Bioreactors serve as enabling technologies that bridge static culture systems and more complex microphysiological models by regulating perfusion, oxygenation, and mechanical loading (Rauh et al., 2011; Leppik et al., 2020; Das et al., 2021; Hao et al., 2021). As early as 2004, bioreactors were recognized as critical tools for driving tissue maturation and mechanotransduction processes that have traditionally been studied *in vivo* (Martin et al., 2004). Common systems, including spinner flasks, rotating wall vessels, and perfusion bioreactors, provide distinct strategies for modulating fluid flow and mechanical stimuli, thereby improving mass transport, cellular infiltration, and osteogenic maturation in engineered constructs (Watson and Mikos, 2023). The controlled addition of physiologically relevant biophysical cues, such as vibrational stimulation, electrical signals, cyclic stretching, dynamic mechanical loading, and fluid shear stress, creates an osteogenic microenvironment that induces MSC differentiation (Rauh et al., 2011; Leppik et al., 2020; Hao et al., 2021; Zhang, 2022; Liu et al., 2026). By recapitulating key aspects of the *in vivo* mechanical environment in a controlled and reproducible manner, these platforms enhance the predictive capacity of *in vitro* systems and extend their potential as Replacement strategies.

While co-culture and dynamic stimulation improve biochemical and mechanical relevance in 2D systems, spatial organization and substrate properties remain critical determinants of musculoskeletal cell function. To address this limitation, micropatterning and topographical engineering approaches have been widely employed to impose physiologically relevant geometry, alignment, and mechanical anisotropy within otherwise accessible 2D culture platforms. Seminal work by Engler et al. in 2006 demonstrated that substrate elasticity alone can bias lineage commitment, with softer substrates (∼1–15 kPa) preferentially supporting myogenic alignment and maturation, and stiffer substrates (∼25–40 kPa and above) promoting osteogenic commitment (Engler et al., 2006; Ragni et al., 2025; Persson et al., 2026). These findings underscore the importance of stiffness-matched, spatially patterned materials for modeling distinct musculoskeletal compartments. Micropatterning and topographical engineering further regulate cell shape, cytoskeletal tension, and alignment, revealing strong links between geometry and fate specification. Nanoengineered substrates incorporating aligned fibers or grooves have similarly been shown to direct morphology and differentiation by mimicking native extracellular matrix (ECM) architecture (Tay et al., 2011; Li and Kilian, 2015; Jenkins and Little, 2019; Cojocaru et al., 2026; Hassan et al., 2026). Together, these strategies substantially extend the utility of 2D systems as partial, and in some cases full, Replacement platforms by capturing essential mechanical and spatial cues governing musculoskeletal biology.

### 4.2 3D *in vitro* culture systems

**Figure 3:**
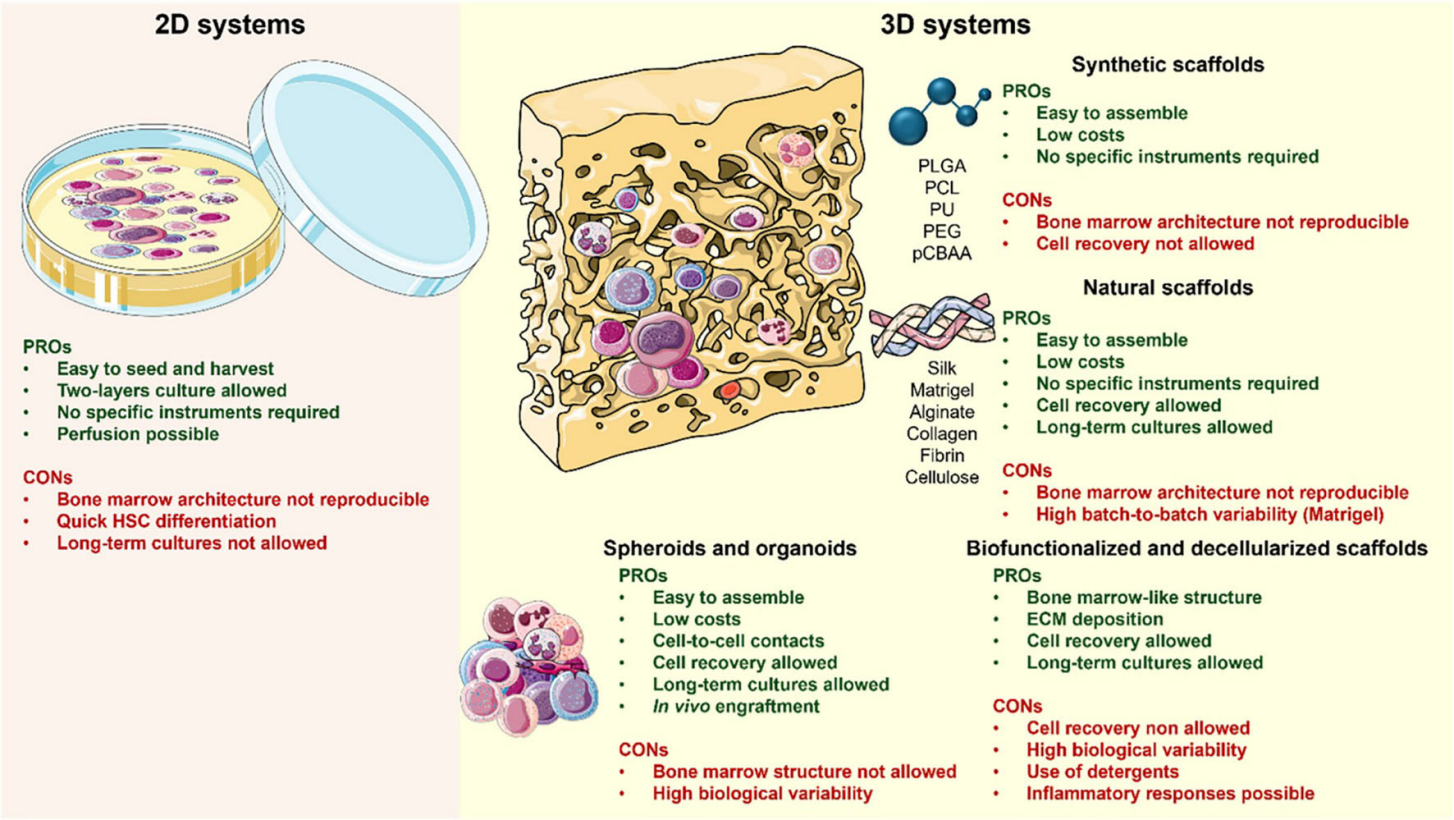
Summary of 2D and 3D in vitro system characteristics for bone marrow tissue modeling. The left panel illustrates 2D culture systems of hematopoietic stem and progenitor cells, typically maintained in well plates or Petri dishes. These systems support controlled exposure to growth factors and cytokines but are limited by short culture duration and reduced physiological complexity. The right panel depicts 3D culture systems incorporating a supporting matrix, enabling heterogeneous cell distribution, proliferation, and differentiation. While these systems better recapitulate native tissue architecture, they are associated with increased variability and challenges in cell recovery. Reproduced from Scala et al., 2026, under the Creative Commons Attribution 4.0 International License (CC BY 4.0) (https://creativecommons.org/licenses/by/4.0/). Figure created using Servier Medical Art (https://smart.servier.com/).

Three-dimensional *in vitro* platforms build on engineered 2D systems by more closely recapitulating the spatial, mechanical, and biochemical context required for native musculoskeletal tissue function (**Figure 3**) (3D In Vitro Models of the Bone Marrow Niche). These systems enable physiologically relevant cell-cell and cell-matrix interactions, collective migration, matrix remodeling, and the formation of physiologically relevant oxygen, nutrient, and mechanical gradients (Nelson and Bissell, 2006; Kapałczyńska et al., 2016; Lelièvre et al., 2017; Cai et al., 2025). By restoring dimensionality and structural context, 3D tissue-engineered models more faithfully reproduce native musculoskeletal architecture and mechanics, positioning them as particularly powerful Replacement strategies for studies that have traditionally relied on animal models.

The 3D *in vitro* systems can be broadly categorized as scaffold-free self-assembling models and scaffold-based, tissue-engineered constructs. Scaffold-free systems, including organoids, rely on bottom-up tissue organization through self-assembly and endogenous matrix production, enabling the recapitulation of key features of bone, cartilage, muscle, and tendon development. These models support high-fidelity investigation of tissue development, disease mechanisms, and therapeutic responses without requiring live animal models, positioning them as a promising Replacement strategy in musculoskeletal research (Liu et al., 2025). For example, Akiva et al. (2021) developed a woven bone organoid in which human BMSCs differentiated into a self-organizing co-culture of osteoblasts and osteocytes, while Iordachescu et al. (2021) seeded osteoblasts and osteoclasts to develop a bone remodeling unit as a trabecular bone organoid.

Conventional organoid differentiation often yields relatively homogeneous structures, motivating the development of modular and hybrid strategies to introduce spatial heterogeneity. Recent strategies such as organoid fusion and microcryogel-supported self-organization have enabled the assembly of distinct musculoskeletal domains, including bi-layered osteochondral organoids with enhanced lineage specification (Lee et al., 2022; Yang et al., 2023). More complex tri-lineage systems, generated by Yin et al., possess self-organized human neuromusculoskeletal organoids from pluripotent stem cells in which neural, muscular, and skeletal lineages emerged simultaneously and formed spatially confined, yet functionally connected domains (Yin et al., 2025). Dynamic, bidirectional crosstalk in organoid systems represent a powerful Replacement platform for studies that have traditionally relied on animal models.

As 3D model complexity increases, substrate composition and spatial architecture become dominant determinants of cell behavior, reflecting the ECM-dependent regulation characteristic of musculoskeletal tissues *in vivo*. Scaffold-based systems allow precise control over matrix chemistry, stiffness, and geometry, enabling systematic evaluation of tissue interfaces. Studies have also identified optimal biomaterial configurations and geometries for stable tendon-bone interfaces, where Alskaykhan et al. identified in 2020 that fibrin hydrogel arrangement could be optimized in a horizontal side-by-side geometry (Alsaykhan and Paxton, 2020).

Beyond discrete compartmentalization, continuous biochemical and structural gradients have been employed to reflect the hierarchical organization of musculoskeletal tissues. Gradient-based scaffolds enable spatially-defined cellular responses across engineered interfaces, capturing gradual transitions in matrix composition and mechanics characteristic of native entheses (Marrella et al., 2016; Vesvoranan et al., 2022). Seidi et al. comprehensively summarized how micro- and nano-fabrication strategies, including hydrogel-based chemical gradients and electrospun fibrous gradients, can be utilized to generate continuous transitions in composition, structure, and stiffness, thereby providing a generalizable toolkit for engineering soft-to-hard interfaces *in vitro* and decreasing reliance on animal models for interface-focused studies *(*Seidi et al., 2011*).* For example, collagen density gradients fabricated on 3D-printed poly(ϵ-caprolactone) (PCL) scaffolds supported region-specific cell behavior across engineered tendon-bone interfaces, illustrating how gradient design principles can model musculoskeletal transitions while decreasing dependence on animal studies (D’Amora et al., 2018). A variety of techniques may be applied in order to recapitulate this process (Caddeo et al., 2017). Chae et al. (2021), for instance, employed spatially graded tendon- and bone-derived decellularized ECM bioinks to induce region-specific differentiation within bioprinted constructs (Chae et al., 2021). Together, gradient scaffold designs and tissue-derived ECM bioinks provide controlled, human-relevant platforms for interrogating interface formation mechanisms that have traditionally required animal models. Recent advancements in 3D printing allow for even more control over scaffold gradients. Alkaissy et al. introduced an innovative manufacturing technique that integrates electrospun filaments with 3D bioprinting to create biphasic scaffolds, mimicking the tendon-bone transition zone (Alkaissy et al., 2022).

Advances in additive manufacturing and bioprinting have further expanded design control, enabling fabrication of musculoskeletal constructs that more closely approximate native tissue architecture. Hybrid fabrication strategies, in particular, combine structural precision with biologically active components to generate spatially organized constructs with compartment-specific functionality (Alcala-Orozco et al., 2021; Huang et al., 2025). In the context of bone tissue engineering, vascularized bone-mimetic hydrogel constructs were generated in 2022 by Sun et al. via PCL 3D printing and gelatin methacrylate (GelMA)-based hydrogel bioprinting to promote tissue formation and organization (Sun et al., 2022). Porosity and compressive properties of the scaffold were adjusted by altering canal numbers and structures, and then canals were seeded with endothelial progenitor cells to support angiogenesis (Sun et al., 2022). This concentric architecture mimics the cortical bone shell and surrounding vascularized marrow space, demonstrating how 3D bioprinting can overcome the limitations of planar culture by enabling compartmentalized, physiologically-relevant bone models *in vitro* (Anada et al., 2019; Zhang et al., 2020; Sun et al., 2022).

### 4.3 Microfluidic and organ-on-a-chip platform

Microfluidic OoC platforms further extend conventional *in vitro* culture by enabling controlled biochemical gradients, fluid shear, and spatial compartmentalization within microphysiological systems. These systems represent one of the most promising emerging strategies for Replacement, as they enable human-cell-based modeling of tissue function, disease progression, and therapeutic response in a highly controlled microenvironment. These platforms typically consist of microscale channels in which engineered microtissues are cultured, with the controlled delivery of small fluid volumes to recapitulate tissue-specific functions and dynamic cellular environments (A guide to the organ-on-a-chip; Scribd; Huang et al., 2022). While early OoC models relied primarily on monolayer cultures, contemporary designs integrate 3D matrices, tissue-specific architectures bioreactors, and organoid systems to create a physiologically relevant system (Diniz et al., 2025).

Recent advances in bone biofabrication within OoC platforms further demonstrate their potential to replace animal models. Microfluidic bone-on-a-chip systems incorporating MSCs and endothelial cells have been used to generate vascularized bone-like tissues, enabling simultaneous investigation of osteogenesis and angiogenesis under controlled flow conditions. Additionally, co-culture systems integrating osteoblasts and osteoclasts within microfluidic environments have successfully recapitulated key aspects of bone remodeling, including cell-cell signaling, migration, and resorption dynamics.

Human musculoskeletal OoCs have emerged as a key Replacement strategy by enabling human-cell-based modeling of tissue function, disease mechanisms, and therapeutic responses without reliance on animal models. Bone-on-a-chip systems leverage microfluidic technologies to recreate the dynamic, multicellular bone microenvironment, offering more physiologically relevant and predictive *in vitro* models for studying bone biology, disease, and therapeutic response than traditional systems (Bone‐on‐a‐Chip, 2021). For example, Zhang et al. utilized a microfluidic system with BMSCs and bone marrow monocytes to model a basic multicellular unit of bone, demonstrating coordinated osteoblast and osteoclast differentiation, recruitment, migration, and signaling. Similarly, interconnected muscle-bone organoids-on-a-chip systems have revealed bidirectional biochemical crosstalk under pathological stimuli (e.g., intermittent hypoxia), demonstrating myokine-mediated regulation of osteogenesis and osteoclastogenesis, and providing mechanistic insights that would otherwise require systemic animal studies (Leung et al., 2022). These bone-on-a-chip systems are also capable of modeling musculoskeletal disorders and enabling the development and evaluation of therapeutic strategies without reliance on animal models (Bone‐on‐a‐Chip, 2021; Lipreri et al., 2023; Lee et al., 2024).

Beyond single-tissue platforms, modular OoC architectures allow bone, cartilage, muscle, tendon, and synovium to be cultured within interconnected microfluidic compartments, enabling controlled investigation of inter-tissue communication while decoupling biochemical signaling from confounding *in vivo* variables. Incorporation of dynamic mechanical loading, perfusion, and real-time sensing further enhances experimental control and translational relevance (Advantages of digital twin technology in orthopedic trauma Surgery – Exploring different clinical use cases). When combined with human primary cells or induced pluripotent stem cell (iPSC)-derived tissues, musculoskeletal OoCs provide scalable, human-relevant platforms that improve prediction of drug safety, efficacy, and pharmacokinetic-pharmacodynamic relationships. For example, Li et al. in 2022 was able to generate a “minijoint” system with the ability to model both a healthy joint system and complex joint diseases (Li et al., 2022). These advanced systems enable high-throughput, fail-early testing strategies, thereby reducing reliance on exploratory animal experimentation while improving translational predictability (Li et al., 2022).

Despite these advances, OoC systems remain limited by challenges in fully recapitulating the complexity of native musculoskeletal tissues, including long-term remodeling, immune system integration, and scalability. Additionally, variability in device design and lack of standardization across platforms can limit reproducibility and widespread adoption. Continued development and validation are therefore required for these systems to fully replace animal models in musculoskeletal research.

### 4.4 Computational tissue simulation

Computational mechanobiological models represent another important class of non-animal approaches capable of Replacing exploratory *in vivo* studies by predicting tissue differentiation and healing outcomes based on physiological cues. These frameworks often integrate mechanical loading, biochemical signaling, and oxygen transport to simulate tissue formation and remodeling across spatial and temporal scales (A computational model that integrates unrestricted callus growth, mechanobiology, and angiogenesis can predict bone healing in rodents; Âon-Alvarado et al; Kumar et al., 2025; García-Aznar et al., 2021; Comellas and Shefelbine, 2022; Perier-Metz et al., 2022b; Bazyar, 2024; Hedayatzadeh Razavi et al., 2024; López-Vaca et al., 2025; Ntousi et al., 2025; Rowe et al., 2025; Vemparala et al., 2025; Kuba et al., 2026). For example, Notermans and Isaksson developed a finite element model (FEM) in 2022 that incorporated both mechano- and oxygen-regulated frameworks to simulate tendon healing and predict the spatial and temporal formation of tendon, cartilage, bone, and adipose-like tissues under mechanical loading conditions (Notermans and Isaksson, 2023). Their predictions captured key experimentally observed features, highlighting the influence of mechanical and oxygenation cues on tissue fate decisions without requiring animal experimentation.

Further, advanced multiscale *in silico* models now integrate mechanical and biological processes to simulate fracture healing and remodeling outcomes, enabling virtual exploration of healing, non-union, disease mechanisms, and therapeutic strategies without extensive animal studies (Multiscale modeling of bone tissue mechanobiology; WIREs Systems Biology and Medicine, 2016; Kameo et al., 2020; Madden et al., 2020; Perier-Metz et al., 2022a; Koller et al., 2024; Marsilio et al., 2025). At smaller length scales, multiscale molecular and continuum frameworks link collagen fibrils, mineral phases, and osteon-level behavior across hierarchical levels, enabling virtual interrogation of parameters that are difficult or impossible to isolate experimentally *in vivo* or *in vitro* (Barkaoui et al., 2016; Sabet et al., 2016; Alijani and Vaughan, 2022).

**Figure 4:**
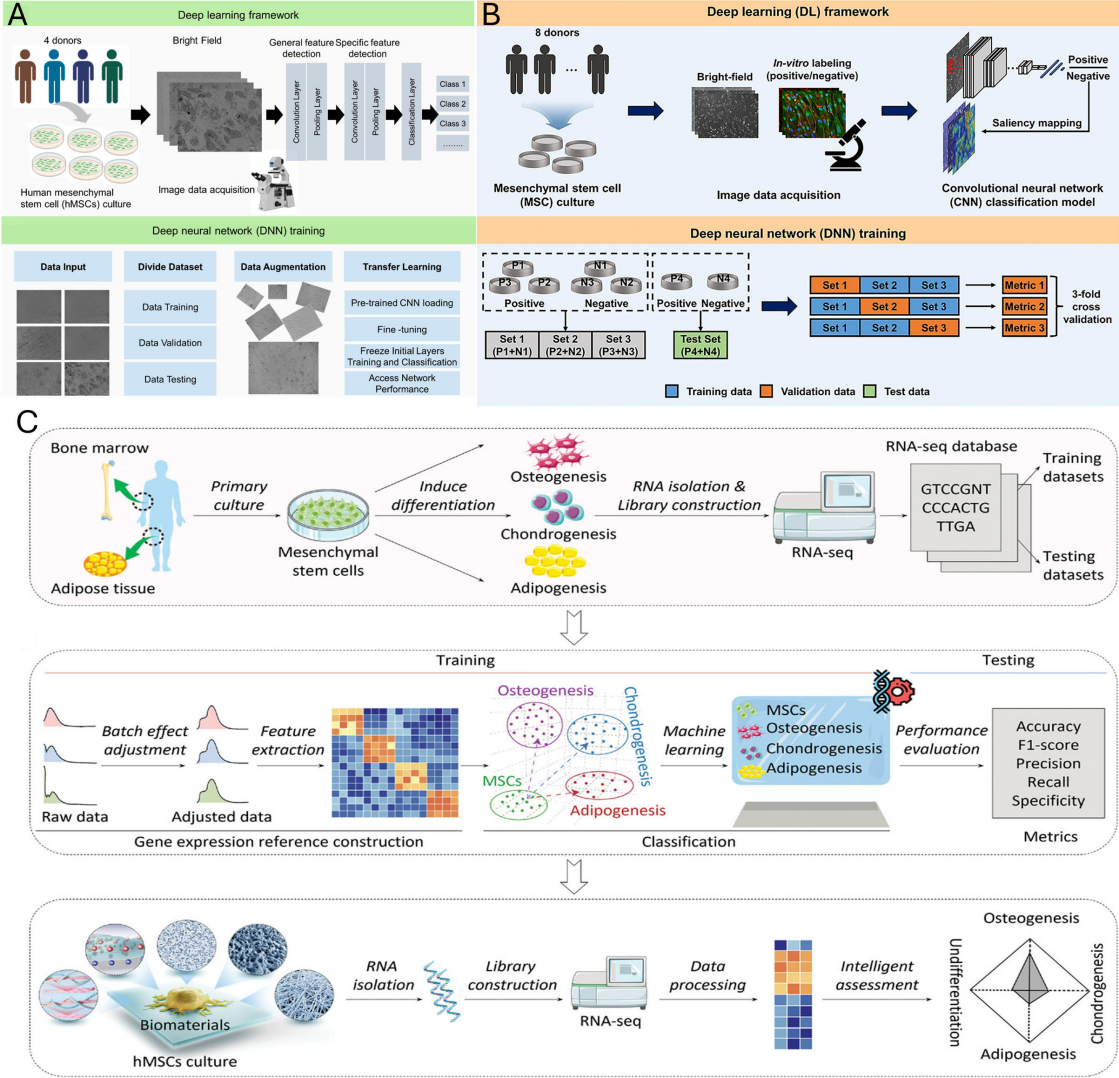
Deep learning and machine learning frameworks for MSC characterization and lineage prediction. **(A)** Schematic of a deep learning and neural network training process used to identify MSC differentiation based on cell morphology. Reproduced from Mai et al. (2023). Copyright © 2023 Mai et al. Distributed under the Creative Commons Attribution License (CC BY) (https://creativecommons.org/licenses/by/4.0/deed.en). **(B)** Schematic of a deep learning framework for functional MSC lineage screening. Reproduced from Kim et al. (2022), licensed under CC BY 4.0 (https://creativecommons.org/licenses/by/4.0/deed.en). **(C)** Overview of an omics-based model for predicting MSC trilineage differentiation. This model utilizes machine learning to predict human MSC (hMSC) lineage fate prior to validation on independent testing datasets. Reproduced with permission from Zhou et al. (2023). Copyright © 2023 Wiley.

Artificial intelligence (AI) is also increasingly integrated within *in silico* and simulative models to improve predictive power, scalability, and translational relevance (Paek et al., 2023; Kolomenskaya et al., 2024; Huang et al., 2025; Shahin and Liu, 2025; Li et al., 2026; Yao et al., 2026). Machine learning models are able to analyze large datasets to predict biomaterial properties, mechanical behavior, tissue-level biomechanics, and therapeutic responses, reducing reliance on traditional experimental screening (Javaid et al., 2023; Fu et al., 2025,). The incorporation of longitudinal physiological data streams into these models represents a promising step toward improving translational relevance. Studies reviewed by Huang et al. in late 2025 used a deep learning framework and deep neural network to identify MSC morphology (**Figure 4**) (Huang et al., 2025; Mai et al., 2023; Zhou et al., 2023; Huang et al., 2025). This and similar tools support Replacement directly by shifting reliance from exploratory animal studies, while also advancing Reduction and Refinement through improved experimental prioritization and fail-early screening (Wu et al., 2024).

However, current AI applications in musculoskeletal research remain constrained by several important limitations. Machine learning models are often trained on static or limited datasets, restricting their ability to capture the dynamic, time-dependent processes that govern tissue development, remodeling, and healing (Jeznach et al., 2024; Wu et al., 2024; Fu et al., 2025). This is particularly challenging in musculoskeletal systems where biological and mechanical cues evolve over time. Recent work has demonstrated that integration of wearable or implantable sensor data with machine learning frameworks enables real-time monitoring and prediction of musculoskeletal healing trajectories, improving model accuracy and enabling adaptive therapeutic strategies (Braun et al., 2025). In addition, many AI models function as “black boxes,” limiting mechanistic interpretability and reducing confidence in hypothesis-driven research and regulatory settings (Wu et al., 2024; Fu et al., 2025).

Digital twin (DT) systems extend computational modeling by integrating multiscale mechanical models with real-time or longitudinal patient data to replicate musculoskeletal structures virtually (Perier-Metz et al., 2022a). By leveraging finite element analysis (FEA), wearable sensor data, and machine learning, DT frameworks can simulate personalized biomechanical responses, predict functional outcomes, and optimize interventions without requiring animal experimentation (Braun et al., 2025). Computational modeling can predict bone fracture healing under varying biological and mechanical conditions (Yang et al., 2024). Applied use cases in orthopedic trauma demonstrate how virtual testing of implant placement, construct stiffness, and loading conditions can narrow the design space prior to clinical translation, thereby Replacing some exploratory animal and preclinical mechanical studies (Barkaoui et al., 2016; Jung and Buehler, 2017).

These computational strategies illustrate how virtual modeling platforms can serve as powerful Replacement tools while simultaneously advancing Reduction and Refinement through improved decision-making and mechanistic insight. Nevertheless, their role in musculoskeletal research remains largely supportive at present, and broader adoption will require continued progress in data standardization, model interpretability, validation, and multiscale integration, particularly given current limitations in capturing patient-specific variability, dynamic healing processes, and complex tissue-tissue interactions.

## 5 Reduction: experimental design and animal use in bone tissue engineering

The second R, Reduction, refers to minimizing the number of animals used in experiments while maintaining scientific rigor and statistical validity. In musculoskeletal and bone tissue engineering research, this principle is particularly important due to the use of invasive defect models, longitudinal healing studies, and variability in regeneration outcomes. One fundamental approach is performing power analyses prior to initiating any animal study. Power analysis uses data from published literature, the significance level (α), and the predicted effect size to calculate the minimum required sample size, ensuring studies are neither underpowered nor use excessive numbers of animals. In bone tissue engineering, appropriate power calculations are especially critical due to variability in defect healing, scaffold integration, and host response across models.

Beyond initial sample size determination, pilot studies can further support Reduction. Small-scale preliminary experiments allow researchers to optimize protocols, identify appropriate outcome measures, and adjust effect size estimates for more accurate power calculations, particularly in bone tissue engineering where variability in defect healing, scaffold integration, and vascularization can impact study outcomes.

Experimental design itself also offers opportunities for Reduction. Factorial study designs can increase informational yield without proportionally increasing animal numbers (Shaw et al., 2002). Similarly, crossover studies can Reduce required sample sizes by limiting inter-animal variability. However, such designs may be difficult to implement in bone tissue engineering where interventions (e.g., defect creation or implant placement) are permanent and not easily reversible. This is especially relevant in critical-sized defect and implant-based models, where repeated interventions are not feasible. Regardless of study type, rigorous randomization and blinding protocols further enhance data quality, decrease bias, and ensure that each animal contributes maximally to scientific knowledge.

### 5.1 Non-destructive assessment techniques

Longitudinal, non-destructive assessment techniques represent particularly effective Reduction strategies, as they allow repeated measurements from the same animals over time. This approach eliminates the need to sacrifice separate cohorts at multiple time points, Reducing overall animal use while increasing statistical power through within-subject comparisons. In the context of bone tissue engineering, several minimally invasive imaging modalities remain underutilized. Among the most promising techniques are micro-computed tomography (microCT), bioluminescence imaging, and ultrasound.

#### 5.1.1 Micro-computed tomography

MicroCT is a well-established, high-resolution imaging modality commonly used *ex vivo* to quantify bone architecture and mineral density (Kim et al., 2021; Shim et al., 2022; Haberthür et al., 2025). Its use for *in vivo* longitudinal imaging, however, remains limited in bone tissue engineering (Namiranian et al., 2025). Live-animal microCT enables real-time tracking of bone healing, particularly in the presence of tissue-engineered scaffolds, eliminating the need for serial sacrifice across time points (Kim et al., 2021; Haberthür et al., 2025). The high-resolution images obtained can be further utilized for micro-FEA analyses to simulate mechanical loading and evaluate bone-scaffold integration (Namiranian et al., 2025). Beyond *in vivo* applications, microCT serves as a powerful characterization tool in scaffold manufacturing, allowing detailed characterization of 3D porous architecture prior to implantation, thereby minimizing the likelihood of advancing suboptimal constructs to animal testing (Olăreț et al., 2021; Ramirez et al., 2024).

#### 5.1.2 Acoustic modal and Periotest analysis

Acoustic modal analysis (AMA), while primarily used for measuring stability of implants, can also be applied to pedicle screws. Instead of measuring fixation through pull-out or dynamic fatigue tests, AMA uses acoustic (sound) response data from an excited mechanical system to assess stability (Einafshar et al). Periotest operates similarly by quantifying the mobility of components on a Periotest Value (PTV) scale ranging from -8 (most stable) to +50 (least stable) (Einafshar et al). While also used frequently in the dental space, Periotest has been verified for grading the quality of osseointegration (Einafshar et al).

#### 5.1.3 Bioluminescence imaging

Bioluminescence imaging is another underutilized yet powerful Reduction tool in bone tissue engineering. This technique involves introducing a bioluminescent reporter gene, typically luciferase, into cells of interest (Logeart-Avramoglou et al., 2010; Wang et al., 2021). Following administration of the substrate (luciferin), an enzymatic reaction produces photons that can be detected and quantified noninvasively using specialized cameras. Because the substrate is rapidly metabolized and causes no harm to the animal, repeated imaging sessions are feasible (Sadikot and Blackwell, 2005).

Transgenic mice expressing luciferase under tissue-specific promoters allow real-time tracking of gene activity. While most commonly employed in cancer research to monitor tumor progression, bioluminescence imaging has considerable potential in bone tissue engineering for assessing inflammation, osteogenic differentiation, apoptosis, and angiogenesis over time (de Boer et al., 2006; Wang et al., 2021). Furthermore, luciferase-labeled cells seeded onto scaffolds can be monitored longitudinally to evaluate survival, differentiation, and migration without requiring interim euthanasia (Wang et al., 2021).

#### 5.1.4 Ultrasound

Ultrasound imaging represents an additional underutilized modality for bone tissue evaluation. Although historically considered difficult to interpret for skeletal tissues, advances in imaging resolution and computational analysis have significantly improved its utility. Ultrasound offers several advantages: it generates no ionizing radiation, has low operational costs, and, with appropriate training, enables rapid image acquisition in longitudinal fracture-healing studies. Modern systems feature intuitive user interfaces, and emerging deep learning algorithms further enhance its potential for automated quantification of healing progression over time (Teng et al., 2023).

### 5.2 Limiting animal tissue waste

Beyond improvements in imaging and experimental design, maximizing the scientific value obtained from each animal represents an important Reduction strategy. Systematic collection of multiple tissue types, beyond the primary study focus and endpoint, allows a single animal to contribute to several complementary research questions. For example, bone tissue engineering studies may simultaneously collect blood for biomarker analysis, contralateral bones for baseline or internal comparisons, and peri-scaffold tissues for inflammation and immune profiling.

Biobanking and tissue-sharing programs further amplify this approach by preserving and cataloging samples for use by other investigators. While implementation of such programs requires institutional infrastructure and may require material transfer agreements, the resulting decrease in total animal use justifies the investment. The Cornell Veterinary Biobank provides one example designed to extend the utility of collected specimens (Cornell Veterinary Biobank). As a practical first step, establishing communication pathways between researchers and veterinary staff to coordinate anticipated sample needs and standardized collection protocols can lay the groundwork for more formalized biobanking initiatives.

To further Reduce animal use, high-throughput *in vitro* screening, utilizing techniques discussed in the Replacement section, should be employed whenever feasible prior to advancing to *in vivo* experimentation. By advancing only the most promising candidates to animal testing, researchers can meaningfully Reduce overall animal numbers while improving experimental efficiency and translational focus.

## 6 Refinement: improving animal welfare and data quality

While Replacement and Reduction aim to limit animal use, Refinement focuses on minimizing pain, distress, and cumulative burden in animals that must still be used. In musculoskeletal research, where invasive procedures and long healing periods are common, Refinement strategies are essential not only for ethical reasons but also for improving data quality and translational reliability. While the following strategies are broadly applicable across animal research, we emphasize considerations particularly relevant to musculoskeletal and bone tissue engineering contexts. Optimized protocols better control variability introduced by stress, pain, infection, or poor recovery, thereby strengthening experimental outcomes (Lang, 2022).

### 6.1 Bone surgery advancements

Advancements within surgical techniques represent one of the most direct Refinement strategies in bone tissue engineering. Optimized surgical planning eases postoperative discomfort, shortens recovery time, decreases infection risk, and improves overall animal wellbeing, all of which contribute to more reliable data.

Recent Refinements in surgical strategy illustrate this principle. Masquelet’s induced membrane technique, for example, preserves and utilizes the periosteum as a biologically active membrane that enhances vascularization and nutrient delivery during bone regeneration (Alford et al., 2021). Developments in bone fixation during surgery are among the most profound advancements found in recent literature. Protocols for optimizing and standardizing osteotomies and distractions in osteogenic rodent models (Jiang et al., 2021; Lin et al., 2024a) and analyses on periprosthetic nailing strategies using locked plating and retrograde intramedullary nailing (Abdallatif and Isaac, 2025) are among the newer techniques being implemented in small animals.

The RISystem, a commercially available device for external fixation, represents a standardized skeletal fixation platform specifically designed for preclinical animal research, enabling highly controlled stabilization and reproducible defect modeling across species (Jiang et al., 2021; Wehrle et al., 2021; Lin et al., 2024b). Such systems minimize surgical variability, improve mechanical consistency during healing, and reduce complications associated with implant instability or inconsistent fixation techniques.

Refinement in this space has also included the development of angle-stable interlocking nailing in a canine femoral CSD model designed to consolidate outcome assessments within a single cohort (Saunders et al., 2022). This fixation strategy allows imaging, biomechanical testing, and histology to be performed in the same cohort of dogs, improving cross-modal data integration and reducing the total number of animals required. These advances highlight how Refinement strategies can simultaneously enhance animal welfare, improve reproducibility, and increase overall experimental efficiency.

### 6.2 Addressing physiology gaps in rodent models

Refinement also includes improving biological relevance to prevent unnecessary or poorly translatable studies. While rodent models remain the most widely used in preclinical research, there is increasing evidence that therapeutic success in rodent models does not consistently translate to clinical outcomes in humans (Gliksten and Yip, 2023; Marshall et al., 2023; Frühwein and Paul, 2025). This discrepancy reflects limitations in cross-species modeling and underscores the need for improved physiological alignment with human disease to restore clinical relevance and prevent wasteful experiments on animals (Lang, 2022).

As discussed, rodent models are less bound by restrictions and documentation requirements in the United States, consequently making mouse and rat models the default starting place for many preclinical experiments. However, most existing rodent (and even larger animal) models are designed to reflect the key features of the bone pathology but fail to replicate conditions as they occur in humans (Shen et al., 2021; Gliksten and Yip, 2023; Marshall et al., 2023; Frühwein and Paul, 2025). While rodent models benefit from fast metabolism and healing rates, which accelerate experimental outcomes in bone healing, their anatomy often lacks the size and complexity required to achieve true translational relevance (Gliksten and Yip, 2023; Marshall et al., 2023; Frühwein and Paul, 2025; Gelles et al., 2025). Additional limitations include simplified disease states, absence of comorbidities, and differences in immune response and remodeling dynamics compared to human systems (Gliksten and Yip, 2023; Marshall et al., 2023). Importantly, early validation in models that more accurately replicate human bone architecture may Reduce total animal use by preventing late-stage translational failure.

### 6.3 Bone marrow ablation, irradiation, and reconstitution

Bone marrow is often accessed either as a part of a larger surgery or for introduction of drugs. Bone marrow ablation and reconstitution procedures are widely used in immunology, oncology, and even aging studies (Hawkins et al., 2025) due to the rich cellular composition of the marrow.

These protocols carry significant welfare implications, including risk of graft-versus-host disease (GVHD), immune suppression, and systemic toxicity. Although legislation requires these procedures to be performed in ways that minimize animal suffering as much as possible, they are still classified as severe (Category E in the European framework; Category D/E in the United States and Canada), reflecting the high potential for pain and distress (Hawkins et al., 2025).

Refinement of irradiation and reconstitution protocols begins with colony and facility logistics. Implementing microbial barrier levels has been shown to have a direct impact on survival by mitigating complications such as swollen muzzle syndrome after radiation exposure (Rios et al., 2022). Best microbial barrier outcomes occur in facilities with ventilated caging, sterilized drinking water, and personnel that implement clean-room personal protective equipment (PPE) (Rios et al., 2022). When transferring living tissues between animals (including genetic material or marrow-derived cells), housing donor and recipient animals within the same facility typically also yields improved results (Hawkins et al., 2025). Introducing living tissues from external environments can increase risk of pathogens or trigger immune responses post-implantation.

Genetic compatibility and strain matter as well. If a genetic duplicate cannot be used, use of same-sex donors and recipients with substantial shared ancestry (e.g., at least 10 generations) is recommended to help prevent allogenic responses and elevated GVHD risk (Ehx et al., 2024; Hawkins et al., 2025). If donor and recipient strains are genetically divergent, the graft becomes allogenic and GVHD risk increases (Ehx et al., 2024; Hawkins et al., 2025). In sex-mismatched transplants, especially female-to-male transplants, chronic inflammation in male reproductive organs may occur, whereas same-sex transplants mitigate this risk (Holler et al; Hawkins et al., 2025). Genetically modified animals may also generally be less tolerant of ablation (Hawkins et al., 2025). Even robust strains can experience adverse effects including nausea, lethargy, off-target cell death from irradiation and/or chemical toxicity, discomfort, and stress associated with engraftment failure, GVHD, or Host-Versus-Graft-Disease (HVGD) (Hawkins et al., 2025; Nowak et al., 2025). Refinement includes tailoring conditioning dose and schedule to each strain’s sensitivity and each animal’s size and condition as well as establishing clear time limitations on murine studies based on their severity category (Nowak et al., 2025).

Finally, technical equipment calibration is a key requirement for Refinement. Routine equipment calibration and pilot studies decrease the risk of mis-dosing due to radiation source decay or unintended energy output differences (Hawkins et al., 2025). Because energy sources and delivery systems vary, even subtle differences can have significant biological and welfare consequences, reinforcing the need for verification and standardized controls (Hawkins et al., 2025).

### 6.4 Anesthesia and analgesia

A central component of Refinement in bone research is optimization of anesthesia and analgesia. Bone tissue is deep within the body and highly innervated, making surgical access inherently painful and necessitating comprehensive perioperative pain management (Magnusdottir et al., 2021). Bone pain can be immediate following skeletal injury or manipulation and may persist as chronic pain in disrupted or delayed healing conditions (Magnusdottir et al., 2021; Iafrate et al., 2022). The nociceptive fibers are readily activated by movement, causing high bone sensitivity during procedures that involve tissue displacement and fixation (Magnusdottir et al., 2021; Iafrate et al., 2022). Therefore, pain assessment and monitoring are vital for current animal studies.

Recent reviews emphasize the need for continued development of pain management strategies in animal experimentation, including improved analgesic approaches that minimize suffering without compromising experimental interpretation (Iafrate et al., 2022; Wolter et al., 2022; Hyndman et al., 2024). Fracture healing studies are especially relevant because post-fracture pain is common and, at present, no intervention fully eliminates pain without potential impacts on bone repair biology (McVeigh et al., 2020). While pain perception is influenced by factors such as age, sex, BMI, genetics, and psychosocial (i.e., sensory, emotional, and perception) factors, baseline analgesia remains a non-negotiable Refinement requirement (McVeigh et al., 2020). Even at the smallest mammal scale, mouse femoral fracture procedures are considered moderately painful, and inadequately controlled pain can impair wound healing, contribute to immunosuppression, and increase risk of infection, each of which can compromise both animal welfare and data quality (Anup et al., 2024).

One study from 2022 by Wolter et al. directly compared tramadol and buprenorphine in a mouse osteotomy model to evaluate efficacy, side effects, and impacts on experimental outcomes (Wolter et al., 2022). Because NSAIDs and Coxibs (COX-2 inhibitors) can both suppress prostaglandin synthesis through cyclooxygenase 2 inhibition, they can impair fracture repair (Park et al., 2019; Wolter et al., 2022). Hence, opiates are often preferred for pain management to avoid interference with bone healing biology (Park et al., 2019; Wolter et al., 2022). Buprenorphine is fast-acting, highly potent, and widely used, whereas Tramadol offers dose flexibility that can support either mild or more severe pain depending on concentration, despite its relatively short half-life (Lang, 2022; Wolter et al., 2022). However, high Tramadol doses have been associated with diminished food and water intake, suggesting adverse effects on wellbeing compared to lower-dose Tramadol and Buprenorphine (Lang, 2022). Closely adhering to dosing recommendations and accounting for drug duration of action can therefore improve humane pain control while also limiting indirect suppression of bone healing associated with stress, poor mobility, or inadequate recovery.

### 6.5 Animal handling and housing best practices

Regardless of whether experimental interventions are surgical, pharmacologic, or environmental, animal handling and interfacing are unavoidable in any *in vivo* animal studies and represent a major opportunity for Refinement.

Handling practices are often inherited culturally across research groups and may receive less routine reassessment than procedures used in larger animal models. Refinement requires updating these practices to reflect evidence-based strategies that mitigate handling stress and improve welfare. Transporting, tagging, restraint, and general handling should be minimized and performed using best practices to avoid unnecessary distress (Rios et al., 2022; Hawkins et al., 2025). The concept of “cumulative severity” captures the total burden of all stressors, medical and routine, experienced by an animal, recognizing that smaller stressors can aggregate into a substantial overall impact. Managing cumulative severity requires reviewing each event the animal experiences and modifying avoidable pain points. For example, irradiation, treatment, or surgery recovery may be performed in the home cage with housing and/or bedding material to prevent extraneous movement due to agitation or anxiety and, when appropriate, even enclosure mates, rather than transferring animals to unfamiliar cages which can provide distraction and reduce self-harming behavior that could jeopardize bone health and/or healing (Hawkins et al., 2025).

**Figure 5:**
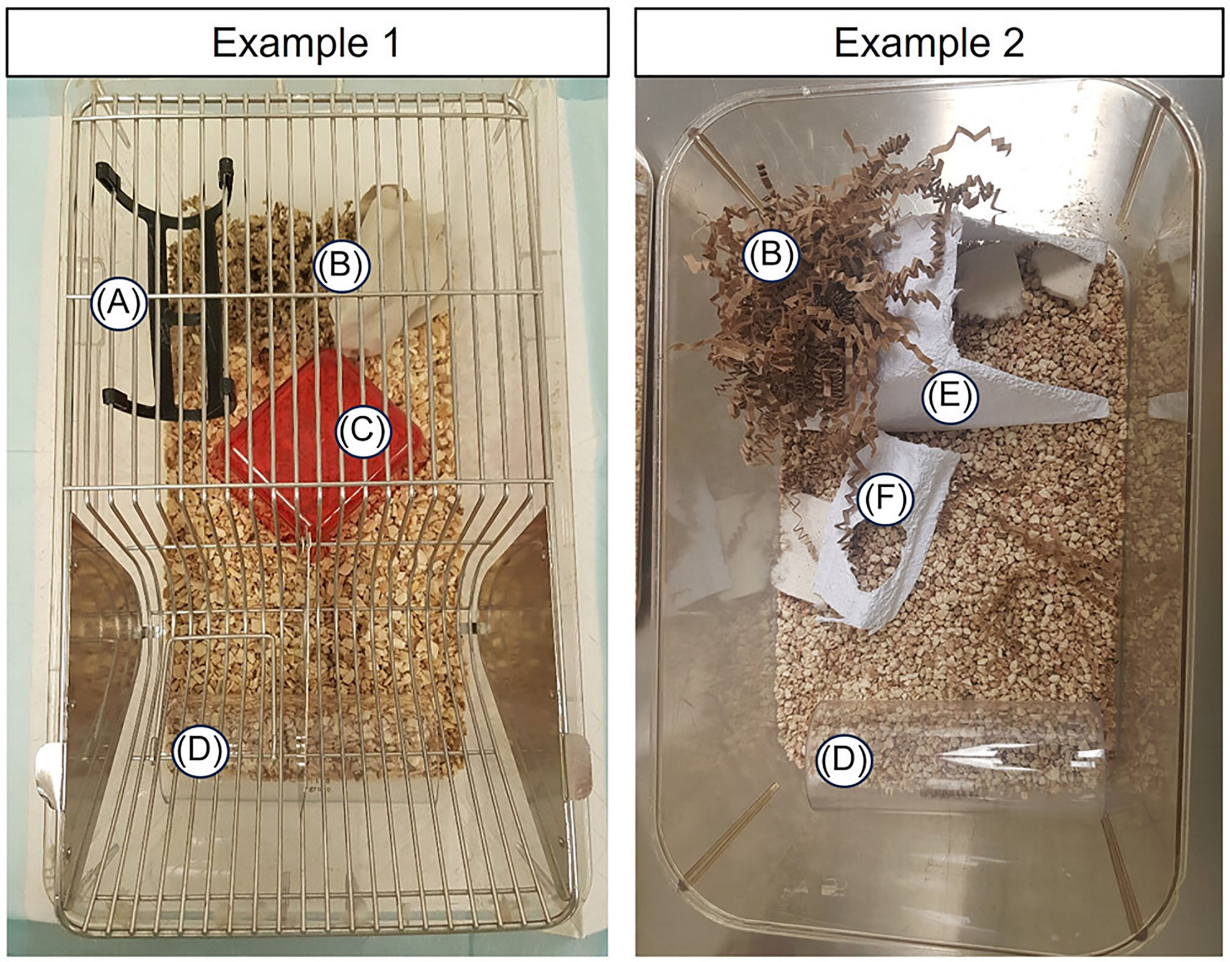
Rodent cage enrichment examples. **(A)** Double swing for multi-level platform options (Datesand); **(B)** heterogenous nesting material allowing for nesting, e.g., Enviro-dri, Nestlet, paper sheets; **(C)** plastic house for shelter and covered huddling; **(D)** clear plastic handling tunnel for minimization of handling-related stress; **(E)** Sheperd Shack (paper house) with an enlarged entrance area to avoid injury with external fixator during recoveries; **(F)** additional cage enrichment, for climbing and gnawing. Reproduced with permission from Anup et al., 2023, Journal of Orthopaedic Research (Wiley). Images © Annemarie Lang.

The living environment is also highly impactful on animal wellbeing and movement allowances which can be imperative in bone outcomes. While specific housing standardizations beyond minimum cage dimensions are not well-outlined, most facilities are adopting enrichment and environmental features which are widely considered as modifications that do not introduce significant drawbacks in standardization (von der Beck et al., 2024). These best practices include improving housing conditions beyond the legal minimum requirements for the target species. Specific recommendations, regardless of species, include providing spaces that allow for postural adjustments (e.g., stretching, sitting, standing) and normal physiological behaviors (i.e., foraging, nesting, grooming, and bathing) which provide physiological loading on the musculoskeletal system according to typical animal movement (Management of Laboratory Animals). In mouse studies especially, this can be accomplished by providing solid flooring, use-appropriate bedding, and sufficient nesting and gnawing material (Rios et al., 2022; von der Beck et al., 2024; Hawkins et al., 2025). Animals should also be housed in compatible social groups to discourage fighting, avoidance, and resource guarding where applicable (Management of Laboratory Animals; Rios et al., 2022; Hawkins et al., 2025). A varied diet with targeted enrichment opportunities (**Figure 5**) (Falcon et al., 2019; Anup et al., 2024), such as multi-level surfaces and shelters, can also ease stress and inter-group conflict while providing opportunities for healthy bone loading and impact (Management of Laboratory Animals; Hawkins et al., 2025). Maintaining appropriate light and sound routines in a temperature-optimized environment can further alleviate stress and support physiologic stability (Management of Laboratory Animals; Hawkins et al., 2025). Finally, scheduling experiments and cage changes thoughtfully, so that disruptive events are staggered and not clustered, may also improve recovery time while limiting stress and cumulative burden (von der Beck et al., 2024; Hawkins et al., 2025).

Working within the musculoskeletal system, especially on bone, means that any surgeries or interventions will likely be highly invasive and require considerable recovery. While normal ranges of motion should be considered and planned for, cages might also need to be modified to allow for a limited range of motion during recovery. Food content and placement may require modification if nausea or mobility changes alter foraging habits (Hawkins et al., 2025). Water access should be monitored closely; bottles may need to be repositioned to ensure reachability, and water should be clean and sterile (Hawkins et al., 2025). Mouse handling itself can also be Refined by replacing tail handling with tunnel handling or cupped hands, both of which decrease stress responses (Hawkins et al., 2025). Habituation, such as tube training or advance exposure to radiation chambers and/or recovery environments, can also moderate stress responses during experimental execution (Hawkins et al., 2025).

Pre-procedural physical examinations before animals are subject to operations or irradiation further Refine welfare and help prevent unexpected losses. Animals should meet predefined baseline health criteria prior to any procedures or irradiation, and these parameters should be monitored regularly throughout the study. Suggested weight benchmarks include ~18g for mature male mice and ~16g for a mature females, with additional guidance for smaller or younger animals as appropriate (Hawkins et al., 2025). Accounting for baseline condition and experimental burden can prevent downstream complications and improve both welfare and data consistency.

### 6.6 Humane endpoints

Experimental design and focus vary widely in bone tissue engineering, and each study may require different parameters for ensuring prioritization of animal welfare. There is a lack of documented general guidelines for best practices in bone research: however, broad study design guidelines can be effectively applied. Especially in cancer research, OBSERVE guidelines provide structured approaches for Refining *in vivo* rodent cancer models and can be adapted for musculoskeletal research (The OBSERVE guidelines provide refinement criteria for rodent oncology models; De Vleeschauwer et al., 2024).

Attentive monitoring and well-defined humane endpoints are essential Refinement practices to prevent lengthy or gratuitous suffering (Danridge et al., 2024). A humane endpoint should be established as a predetermined limit for acceptable distress, at which point the animal’s suffering must be addressed or ended within the constraints of the study (The OBSERVE guidelines provide refinement criteria for rodent oncology models; Rios et al., 2022; De Vleeschauwer et al., 2024; Hawkins et al., 2025). Indicators can include significant weight loss, visible signs of illness, adverse and prolonged behavioral changes, hypothermia (including thermal camera detection), and facial grimacing (De Vleeschauwer et al., 2024; Hawkins et al., 2025). Pain, in particular, is common in musculoskeletal interventions and signs of physical discomfort should be monitored.

Specific symptoms can also indicate severe complications requiring immediate action, and may necessitate a humane ending (Hawkins et al., 2025). For example, diarrhea, skin lesions, frailty, and anemia 2–3 weeks post-procedure my indicate GVHD. Hunched posture, lethargy, dehydration, hyperventilating, and failure to self-groom are also common signs of sickness while minimal responsiveness to external stimuli and swollen oral mucosa are more severe distress signs (Hawkins et al., 2025).

### 6.7 Documentation and reporting standards

Documentation is a critical and often underappreciated component of Refinement. Even in large-animal studies, strain information is frequently underreported despite evidence that animal strain can influence experimental outcomes (Göpel and Burggren, 2022; Sillmann et al., 2025). Bone research also presents substantial heterogeneity in study designs, and CSDs are often implemented without species- and site-specific definitions (Significance and considerations of establishing standardized critical values for critical size defects in animal models of bone tissue regeneration, 2024; Wei et al., 2024; Sillmann et al., 2025).

A 2025 review by Sillmann et al. on mandibular defect models reported that among 27 papers reviewed for risk assessments, none provided detailed study protocols or included clear allocation/randomization processes (Sillmann et al., 2025). The reviewed studies also lacked reporting on dropout and substitute management, or blinding processes of caregivers and outcome assessors (Sillmann et al., 2025). While these gaps do not directly translate to the treatment of the animals, they raise concerns about bias and irreproducibility, both of which can drive repeated or unnecessary animal use (Wilson et al., 2023). As of 2020, compliance for reporting important variables surrounding animal experimentation was still hovering around ~60-65% (Göpel and Burggren, 2022).

To address this, ARRIVE guidelines provide a standardized checklist and informational resource with an established structure for transparent reporting and reproducible descriptions of *in vivo* experiments (Wilson et al., 2023; Sillmann et al., 2025). First introduced in 2010 and updated as ARRIVE 2.0, these guidelines outline best practices to improve rigor, reproducibility, and interpretability of animal studies (The ARRIVE guidelines 2.0 | ARRIVE Guidelines; Percie du Sert et al., 2020). Standardized reporting strengthens welfare oversight, improves the value derived from each animal study, and decreases downstream waste (Diederich et al., 2022).

### 6.8 Strategic animal selection

Beyond procedural Refinement, model selection itself is an important Refinement decision for improving efficacy and efficiency of bone tissue engineering studies (Chang and Grieder, 2024). Ethical concerns around animal use are compounded by limitations in physiological relevance, particularly in bone tissue, which is highly size- and load-dependent (Ribitsch et al., 2020). Careful model selection, including pathology-relevant age ranges, sex inclusivity, and strain analysis (Hawkins et al., 2025), can support clinical translation. Thoughtful selection decisions made early in study design improve both welfare and translational relevance by avoiding unnecessary or poorly predictive studies.

Regulatory bodies including the Federal Food and Drug Administration (FDA) and the European Medicines Agency (EMA) recommend a two-stage approach to animal models: i) smaller laboratory animals for early screening and development, and ii) larger animal models for evaluation of efficacy, host response, degradation, and longer-term outcomes (Ethical use of animals in medicine testing; Commissioner O of the Animal Welfare; Ribitsch et al., 2020; Kiani et al., 2022). Determining when escalation to a large-animal study is warranted can be difficult, but alignment of model choice to the biological question is essential to avoid waste. For example, moving an untested bone scaffold material into an ovine model may be convenient for surgical workflow but may also be wasteful if foundational biocompatibility and proof-of-concept testing have not been performed. Conversely, repeatedly testing a scaffold or other bone intervention in murine models may provide limited value if biomechanical loading and scale cannot approximate the intended clinical context. By implementing this approach, the number of laboratory animals will not only be Reduced, but the quality of the research outcomes will also be improved.

Additionally, when selecting models for an animal study, natural disease models represent another often-overlooked Refinement opportunity. Purposeful use of less-radiation-resistant strains may be required to achieve bone radiation and cancer study outcomes; however, such decisions should be accompanied by a harm-benefit analysis that accounts for increased animal suffering and the need for enhanced monitoring and care (Hawkins et al., 2025).

Spontaneously occurring injuries or diseases that mirror human musculoskeletal pathologies may provide more clinically relevant data than induced models. For example, animals with naturally occurring osteoporosis may better reflect human osteoporosis than many experimentally-induced models (Ribitsch et al., 2020).

This perspective aligns with the “One Health One Medicine” framework, which recognizes shared mammalian biology and pathology patterns, and can improve predictive validity when appropriate models are available (Ribitsch et al., 2020). This approach requires careful selection of animals based on biological similarity, epidemiology, outcome measures, and availability of relevant diagnostic tools. In the musculoskeletal space, using animals with naturally-developed bone pathologies can help temper some of the challenges of preclinical animal work.

### 6.9 Limiting animal tissue waste

Advances across the clinical and preclinical research space have accelerated development of methods that support Replacement, Reduction, and Refinement. Organoids, additive manufacturing and bioprinting, artificial intelligence (AI) and machine learning, and OoC technologies are collectively expanding the toolkit for human-relevant modeling while diluting reliance on animal experimentation.

However, within *in vivo* studies, maximizing the value of each animal remains a critical Refinement strategy. Systematic collection of multiple tissue types, integration of multimodal analyses, and coordinated sample sharing can significantly reduce the total number of animals required across studies. These approaches ensure that each animal contributes maximally to scientific knowledge while minimizing unnecessary duplication of experiments.

### 6.10 Organoids, organ-on-a-chip, and self-assembly of tissue hierarchy *in vitro*

Bone is structurally and biologically complex, with dynamic remodeling and multiple functional compartments, making it challenging to recapitulate using current organoid strategies (3D bioprinting bone/cartilage organoids: construction, applications, and challenges). Due to the spatially demanding nature of the target tissue architecture, bone organoids are especially hindered by size limitations. Larger organoids quickly encounter limitations in oxygen diffusion, and immune regulation critical for bone maintenance has not yet been effectively simulated (3D bioprinting bone/cartilage organoids: construction, applications, and challenges; Huang et al; Hong et al., 2025; Wu et al., 2025). Moreover, both osteogenic development and remodeling processes require coordinated activity among osteoblasts, osteoclasts (Huang et al), and supporting stromal populations, which often exhibit limited proliferative capacity and can be difficult to sustain and organize into stable, self-patterning structures (3D bioprinting bone/cartilage organoids: construction, applications, and challenges).

**Figure 6:**
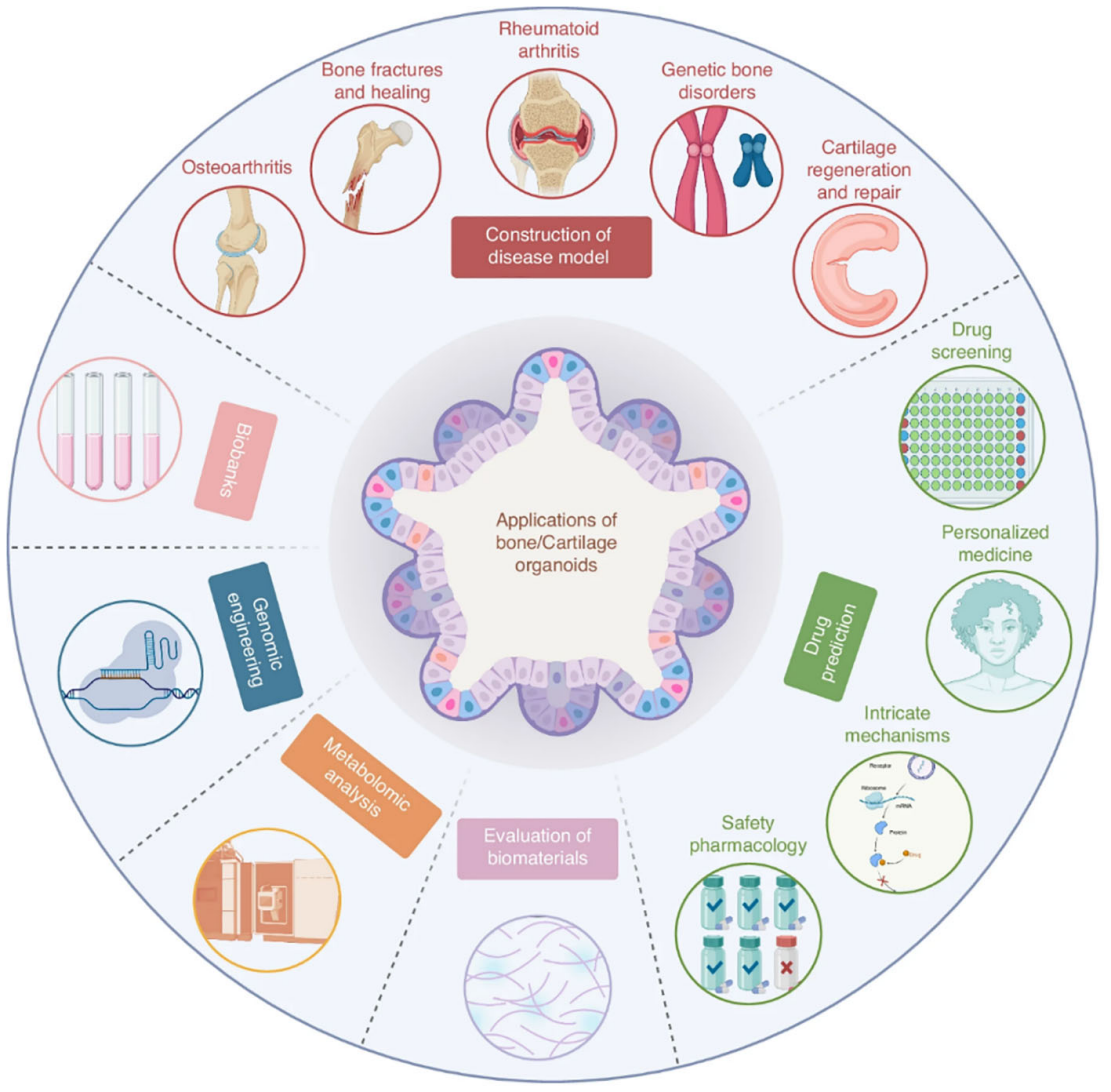
Summary of applications of bone and cartilage organoids. Applications are organized within a circular schematic, with physiological and pathological modeling shown in the upper portion, and drug screening and pharmacological applications in the lower right section. The remaining segment highlights additional application areas, including bioink development, genomic engineering, metabolomic analysis, and biomaterial characterization. Reproduced from Bai L. et al., 2024 (Bone Research), licensed under CC BY 4.0 (https://creativecommons.org/licenses/by/4.0/deed.en).

Despite these challenges, MSCs provide a promising entry point for bone organoid development and vascularization strategies (3D bioprinting bone/cartilage organoids: construction, applications, and challenges; Hong et al., 2025). Embryonic MSCs have the highest proliferative and differentiative abilities, but come with ethical concerns associated with embryonic tissue use (3D bioprinting bone/cartilage organoids: construction, applications, and challenges; Huang et al). Alternatively, iPSCs, mesenchymal stromal cells from bone marrow, umbilical cord, placenta, and adipose tissue can also be used for their osteochondrogenic potential (3D bioprinting bone/cartilage organoids: construction, applications, and challenges). Bone organoids hold considerable promise for applications in a wide range of disease modeling and regenerative studies, which would otherwise require highly invasive animal models (**Figure 6**). Accordingly, significant effort has been directed toward developing viable and reproducible bone organoid platforms (Bai et al., 2024; Zhao et al., 2024).

Approaches to organoid assembly vary substantially. Organoids can be formed using microfluidic strategies that engineer microchannels and chambers to guide tissue structure and microenvironmental cues, an approach that can naturally integrate with OoC technology (3D bioprinting bone/cartilage organoids: construction, applications, and challenges; Lei et al; Hu et al., 2023). Alternatively, organoids may rely on self-organization, where stem cells are exposed to defined environmental signals that encourage intrinsic patterning into tissue-like architectures (Lei et al; Yin et al., 2025).

Nevertheless, current organoid assembly methods cannot yet reliably reconstruct the highly organized bone microarchitecture. Key features such as Haversian canals, lamellae, lacuna, and embedded osteocytes remain difficult to reproduce within existing platforms and length scales (3D bioprinting bone/cartilage organoids: construction, applications, and challenges). In addition, physiologically meaningful bone models require vascularization beyond diffusion limits; as organoid size increases, passive diffusion is unlikely to meet metabolic demands without integrated perfusion strategies (3D bioprinting bone/cartilage organoids: construction, applications, and challenges).

The growth and development of bone-on-a-chip models have progressed, yet at a slower rate in comparison to their soft tissue counterparts. Recent advances in bone biofabrication within OoC platforms further highlight their potential as Replacement strategies. Microfluidic bone-on-a-chip systems incorporating MSCs and endothelial cells have been used to generate vascularized bone-like tissues, enabling simultaneous investigation of osteogenesis and angiogenesis under controlled flow conditions. Additionally, co-culture systems integrating osteoblasts and osteoclasts within microfluidic environments have successfully recapitulated key aspects of bone remodeling, including cell-cell signaling, migration, and resorption dynamics. These approaches demonstrate an increasing ability to model complex, multicellular bone processes *in vitro* while reducing reliance on *in vivo* experimentation.

As mentioned in Section 4 of this paper, this is largely due to the complexity of bone as a composite tissue composed of intricate structural features. However, several recent studies have continued to broaden the utility of bone-on-a-chip models in research. In 2021, a research group developed a polyacrylic-based, laser engraved microfluidic device containing both osteogenic and endothelial phenotype cells, with shear stress enabled via magnetic actuation and media flow to support cell differentiation (Das et al., 2021). The key finding from this study was that biophysical stimuli (e.g., strain, magnetic fields) can act as an alternative to growth factors to support distinct cellular niches required for modeling complex organ systems, with similarities observed to intramembranous ossification. Another research study investigated the development of a structurally relevant model by taking a 3D polymer model of trabecular bone and coating it with hydroxyapatite prior to integration in a microfluidic device for cell culture (Bone‐on‐a‐Chip, 2022). This model recapitulated the porosity and surface morphology of trabecular bone and supported long-term culture of hMSCs. A more recent study investigates a chip system capable of coculturing osteoblasts and osteoclasts to mimic bone remodeling at the level of a basic multicellular unit (Zhang et al., 2025). This model was also used to verify the clinical antiresorptive effects of two anti-osteoporosis drugs.

### 6.11 Bioprinting and manual assembly for high-precision recapitulation of tissue hierarchy *in vitro*

**Figure 7:**
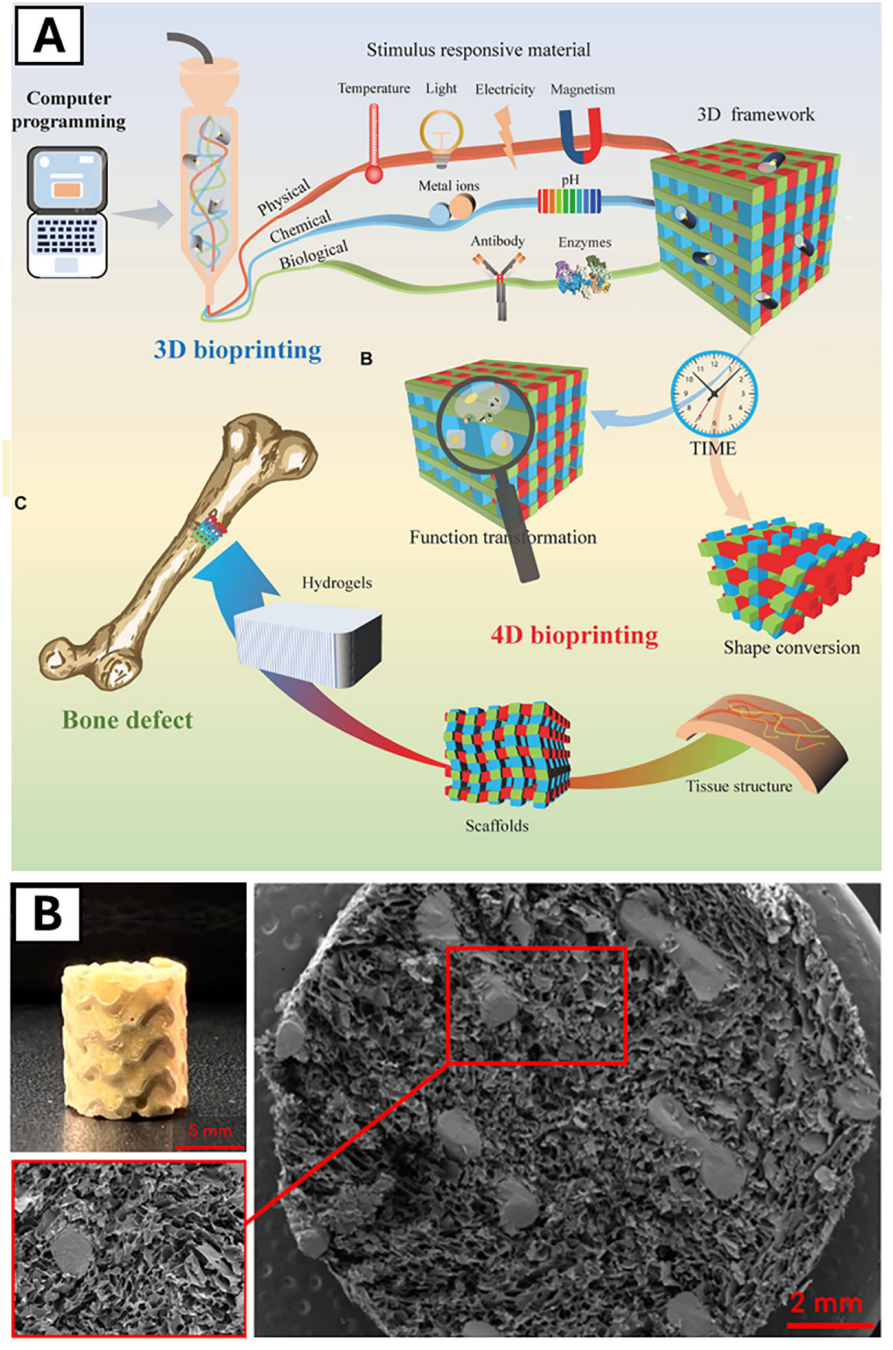
Bioprinting and manual assembly strategies for recapitulating tissue hierarchy *in vitro*. **A)** Highlights of the bioprinting process using physical, chemical, and/or biologically stimulable biomaterials to create a 3D framework. Time dependent changes in scaffold structure represents 4D bioprinting, resulting in an adaptable system capable of dynamically changing in response to the environment it is placed in. Reproduced from Kang X. et al., 2022 (Frontiers in Bioengineering and Biotechnology), licensed under CC BY 4.0 (https://creativecommons.org/licenses/by/4.0/deed.en). **B)** Macroscale view of a combined scaffold featuring 3D printed gyroid integrated into a cryogel matrix. SEM images depict the horizontal cross section (19×) with a zoom in (100×). Adapted from Olevsky L.M. et al., 2023 (Bioengineering), licensed under CC BY 4.0 (https://creativecommons.org/licenses/by/4.0/deed.en).

Bioprinting and manual assembly remain promising strategies for creating *in vitro* bone models because they enable control over geometry, spatial composition, and multicellular organization. Due to its high versatility and ability to fabricate complex architectures, bioprinting has the potential to recreate aspects of biological hierarchy using cell-loaded bioinks (3D bioprinting bone/cartilage organoids: construction, applications, and challenges; Huang et al; Wu et al., 2025). In doing so, bioprinting harnesses two key advantages of complementary technologies. First, it maintains the high specificity and tunability characteristic of traditional 3D printing (3D bioprinting bone/cartilage organoids: construction, applications, and challenges; Huang et al). Second, it enables the incorporation and spatial localization of living cells within defined microenvironments (3D bioprinting bone/cartilage organoids: construction, applications, and challenges; Huang et al; Wu et al., 2025). This blend of high precision paired with scalable, automatable construction supports the long-term promise of bioprinting as a Replacement-oriented strategy for early-stage testing and mechanistic investigation. In addition to bioprinting precision and its ability to reconstruct complex architectures, bioprinting also supports a fourth dimension: time, allowing printed structures to dynamically change shape in response to specific stimuli. Additional scaffold-enabled approaches include electrospinning (to generate fibrous microenvironments via high-voltage fiber deposition) (3D bioprinting bone/cartilage organoids: construction, applications, and challenges) and 3D printing (to manufacture structures layer-by-layer with programmed geometry) (Huang et al; Hu et al., 2023; Hong et al., 2025). These scaffolds can be used to guide self-organization, support targeted cell seeding, or impose microstructural features that are difficult to achieve through self-assembly alone (**Figure 7**) (3D bioprinting bone/cartilage organoids: construction, applications, and challenges; Huang et al; Lei et al; Hu et al., 2023; Hong et al., 2025; Wu et al., 2025).

A major limitation is that bioprinting materials are frequently constrained to relatively soft hydrogel formulations (3D bioprinting bone/cartilage organoids: construction, applications, and challenges; Wu et al., 2025). This presents challenges in bone engineering contexts, where mechanical mismatch between soft gels and mineralized bone can limit structural fidelity and functional relevance. Hydrogels commonly used for bone-focused bioprinting often lack sufficient mechanical strength to properly simulate bone tissue behavior (3D bioprinting bone/cartilage organoids: construction, applications, and challenges; Kang et al., 2022; Maresca et al., 2023; Wu et al., 2025). Encouragingly, recent work has improved mechanical performance by implementing crosslinking strategies to induce mechanical property changes after printing (3D bioprinting bone/cartilage organoids: construction, applications, and challenges). However, mechanical heterogeneity remains a challenge in many cases (Zhao et al., 2024). To address this limitation, complementary strategies have included the integration of supportive lattice structures and the development of shape-retentive cryogels that better tolerate handling and mechanical loading (Bertone et al; Olevsky et al., 2023).

Hybrid and combinatory fabrication strategies further expand the ability to recreate complex biological structures and tissue transition zones. While bioprinting alone is often constrained to soft materials, hybrid and combinatory fabrication strategies enable more physiologically relevant multi-tissue environments. Bone is highly dynamic and relies heavily on its supporting tissues at interfaces; as a result, strategies that modify not only the bone-mimetic scaffold but also the surrounding microenvironment may be particularly important for modeling both healthy and diseased states. For example, scaffolds designed for bone-anchored implants increasingly incorporate natural protein additives to promote cellular adhesion and lessen implant loosening (Cagle et al., 2025). Fabrication techniques such as electrospinning and cryogelation can be combined to address soft-to-hard tissue interfaces (Anup et al., 2026). Dual-material and hybrid scaffolds can also overcome limitations of organoid self-assembly by enabling larger-scale constructs with controlled compartmentalization and interface design. Additionally, the ability to incorporate engineered or tissue-derived proteins can support more physiologically relevant signaling dynamics. A 2021 study by Seims et al. described methods to localize, bind, and mimic growth factor behavior in bone, cartilage, and osteochondral contexts, underscoring how biochemical engineering can complement structural design in advanced *in vitro* platforms (Seims et al., 2021). Heterogeneous tissue interactions and cross-talk have also been investigated in the musculoskeletal space with a 2025 study creating neuromusculoskeletal organoids from embryonic and iPSCs (Yin et al., 2025). Yin et al. demonstrated inter-tissue synergy among the engineered components using transcriptomic analysis and confirmed the development of distinct skeletal regions within their tri-tissue organoid (Yin et al., 2025).

Despite rapid progress, these technologies are not yet sufficient to fully Replace animal models in bone tissue research. Many complex tissue-tissue interactions remain difficult to reproduce, and immune responses, which are critical drivers of healing, remodeling, and implant integration, are still challenging to model robustly *in vitro*. Without more complex representation of bone and its supporting systems, animal studies remain necessary for many translational questions. However, the gap continues to narrow. Continued advances in printable cell suspensions, highly tunable biomaterials, protein engineering, and microfluidic/bioreactor design are steadily moving *in vitro* building blocks closer to the complexity and predictive value of *in vivo* systems.

## 7 Conclusions

The 3Rs framework—Replacement, Reduction, and Refinement—was introduced nearly 70 years ago as a call to recognize and minimize the ethical costs of animal experimentation. Although foundational in hindsight, it represents a relatively recent development within the broader history of modern science. Even more recent was the codification of the 3Rs into actionable legislative and regulatory mechanisms, with many formal requirements emerging only in the 1990s (Grimm et al., 2023).

The aspirational future of science in which animal suffering is not required to drive human health advancement has yet to be realized. Given current biological and technical limitations, fully eliminating animal use in musculoskeletal, particularly bone, tissue engineering is not imminent. However, the pace of innovation over the last century provides strong reason to expect continued progress.

Adoption of the 3Rs remains imperative, and in some jurisdictions is enforced through binding legislation, including the European Directive established in 2010 (Grimm et al., 2023). Organoids, bioprinting and additive manufacturing, and OoC models offer substantial promise, but they remain far from fully Replacing animals across the range of musculoskeletal questions. For the foreseeable future, these platforms are best positioned to supplement animal work by enabling earlier-stage screening, mechanistic discovery, and human-relevant modeling of specific pathways, thereby improving fail-early decision-making before escalation to *in vivo* studies.

As non-animal models mature, they can increasingly Reduce the number and scope of exploratory animal experiments while improving translational relevance. Therefore, current preclinical animal work, particularly once foundational feasibility is established *in vitro*, should continue to prioritize Reduction and Refinement. Animal use may remain unavoidable for many research questions, but the scope and numbers of animals used in *in vivo* studies are highly modifiable.

Rigorous power analysis, careful use of prior literature, and maximizing scientific yield per animal (e.g., longitudinal imaging and multi-tissue sampling) can meaningfully decrease animal use. When animals cannot be fully eliminated, minimizing pain, distress, and cumulative burden is essential not only for ethical reasons but also for scientific validity. Poor welfare and high stress have been shown to degrade data quality and bias outcomes (Anup et al., 2024). This concern is especially pronounced in bone research, which frequently involves invasive surgery and prolonged recovery.

Implementing best practices (e.g., optimizing analgesia, limiting handling stress, defining humane endpoints, and practicing transparent reporting) supports improved animal wellbeing while strengthening rigor and reproducibility (Ethical use of animals in medicine testing; The ARRIVE guidelines 2.0 | ARRIVE Guidelines; Commissioner O of the Animal Welfare; Percie du Sert et al., 2020; Ribitsch et al., 2020; Hawkins et al., 2025). The 3Rs therefore contribute not only to the moral integrity of scientific research, but also to the reliability and interpretability of animal-derived data.

Bone tissue engineering poses unique challenges due to the structural complexity of bone and the mechanical scale required to model clinically relevant loading; nonetheless, clear and feasible steps exist to begin to Replace *in vivo* studies, Reduce animal numbers when necessary, and Refine protocols to improve both ethical and scientific outcomes.

## References

[B13] 3D bioprinting bone/cartilage organoids: construction, applications, and challenges. ScienceDirect. doi: 10.1016/j.jot.2025.08.008 PMC12451290 40989070

[B130] A computational model that integrates unrestricted callus growth, mechanobiology, and angiogenesis can predict bone healing in rodents. Sci. Rep. doi: 10.1038/s41598-024-80502-2 PMC11608358 39616231

[B181] Abdallatif A. Isaac S. (2025). Locked plating versus retrograde intramedullary nailing in the surgical fixation of periprosthetic supra-condylar knee fractures: An updated systematic and meta-analysis review. OBM Geriatr. 9, 1–9. doi: 10.21926/obm.geriatr.2502315

[B126] Advantages of digital twin technology in orthopedic trauma Surgery – Exploring different clinical use cases. Sci. Rep. doi: 10.1038/s41598-025-04792-w PMC12144170 40481259

[B53] Aguirre-Flores M. E. Tokach L. E. Cheang E. Trahan G. Webster D. A. Watson A. L. . (2026). Development of a large porcine model of osteogenesis imperfecta type I. Bone Rep. 28, 101897. doi: 10.1016/j.bonr.2026.101897. PMID: 41626399 PMC12860256

[B94] Ajalik R. E. Alenchery R. G. Cognetti J. S. Zhang V. Z. McGrath J. L. Miller B. L. . (2022). Human organ-on-a-chip microphysiological systems to model musculoskeletal pathologies and accelerate therapeutic discovery. Front. Bioeng. Biotechnol. 10. doi: 10.3389/fbioe.2022.846230. PMID: 35360391 PMC8964284

[B80] Akiva A. Melke J. Ansari S. Liv N. van der Meijden R. van Erp M. . (2021). An organoid for woven bone. Adv. Funct. Mater. 31, 2010524. doi: 10.1002/adfm.202010524. PMID: 41531421

[B89] Alcala-Orozco C. R. Cui X. Hooper G. J. Lim K. S. Woodfield T. B. F. (2021). Converging functionality: Strategies for 3D hybrid-construct biofabrication and the role of composite biomaterials for skeletal regeneration. Acta Biomater. 132, 188–216. doi: 10.1016/j.actbio.2021.03.008. PMID: 33713862

[B178] Alford A. I. Nicolaou D. Hake M. McBride‐Gagyi S. (2021). Masquelet’s induced membrane technique: Review of current concepts and future directions. J. Orthop. Res. 39, 707–718. doi: 10.1002/jor.24978. PMID: 33382115 PMC8005442

[B149] Alijani H. Vaughan T. (2022). A multiscale finite element investigation on the role of intra- and extra-fibrillar mineralisation on the elastic properties of bone tissue. J. Mech. Behav. Biomed. Mater. 129, 105139. doi: 10.1016/j.jmbbm.2022.105139. PMID: 35248874

[B121] Alkaissy R. Richard M. Morris H. Snelling S. Pinchbeck H. Carr A. . (2022). Manufacture of soft-hard implants from electrospun filaments embedded in 3D printed structures. Macromol. Biosci. 22, 2200156. doi: 10.1002/mabi.202200156. PMID: 36048528

[B62] Allen S. L. Elliott B. T. Carson B. P. Breen L. (2023). Improving physiological relevance of cell culture: the possibilities, considerations, and future directions of the ex vivo coculture model. Am. J. Physiol. Cell Physiol. 324, C420–C427. doi: 10.1152/ajpcell.00473.2022. PMID: 36571441 PMC9902212

[B28] Alonso-Fernández I. Haugen H. J. Nogueira L. P. López-Álvarez M. González P. López-Peña M. . (2024). Enhanced bone healing in critical-sized rabbit femoral defects: Impact of helical and alternate scaffold architectures. Polymers 16, 1243. doi: 10.3390/polym16091243. PMID: 38732711 PMC11085737

[B119] Alsaykhan H. Paxton J. (2020). Investigating materials and orientation parameters for the creation of a 3D musculoskeletal interface co-culture model. Regener. Biomater. 7, 413–425. doi: 10.1093/rb/rbaa018. PMID: 32793386 PMC7415002

[B91] Anada T. Pan C.-C. Stahl A. M. Mori S. Fukuda J. Suzuki O. . (2019). Vascularized bone-mimetic hydrogel constructs by 3D bioprinting to promote osteogenesis and angiogenesis. Int. J. Mol. Sci. 20, 1096. doi: 10.3390/ijms20051096. PMID: 30836606 PMC6429349

[B59] Animal Use Alternatives (3Rs) ( National Agricultural Library). Available online at: https://www.nal.usda.gov/animal-health-and-welfare/animal-use-alternatives.

[B204] Anup A. Dieterich S. Oreffo R. O. C. Dailey H. L. Lang A. Haffner-Luntzer M. . (2024). Embracing ethical research: implementing the 3R principles into fracture healing research for sustainable scientific progress. J. Orthop. Res. 42, 568–577. doi: 10.1002/jor.25741. PMID: 38124294

[B239] Anup A. Men M. Wasacz K. Bok M. Limberg A. K. Hixon K. R. (2026). Silk cryogel and electrospun scaffold characterization for bone-tendon interface applications. Front. Bioeng. Biotechnol. 14. doi: 10.3389/fbioe.2026.1685458. PMID: 41923877 PMC13036107

[B132] Âon-Alvarado D. A. G. Duque-Daza C. A. Vaca-González J. J. Casas E. B. L. Linero D. L. de Boer G. . Computational model of bone remodeling integrating osteocyte mechanotransduction and microdamage-driven self-repair. doi: 10.1007/s10441-025-09505-4 41171356

[B5] Stein M. Elefteriou F. Busse B. Fiedler I. A. Kwon R. Y. Farrell E. . (2023). Why animal experiments are still indispensable in bone research: a statement by the european calcified tissue society. J. Bone Mineral Res. 38 (8), 1045–1061. doi: 10.1002/jbmr.4868 PMC10962000 37314012

[B214] (2024). Significance and considerations of establishing standardized critical values for critical size defects in animal models of bone tissue regeneration. Heliyon 10, e33768. doi: 10.1016/j.heliyon.2024.e33768. PMID: 39071581 PMC11283167

[B112] (2026). 3D *In Vitro* Models of the Bone Marrow Niche ( ACS Biomaterials Science & Engineering). doi: 10.1021/acsbiomaterials.5c01421 PMC12801197 41384609

[B122] A guide to the organ-on-a-chip. Nat. Rev. Methods Primers. doi: 10.1038/s43586-022-00118-6

[B3] The New York Declaration on Animal Consciousness. Available online at: https://sites.google.com/nyu.edu/nydeclaration/declaration (Accessed February 12, 2026).

[B211] The OBSERVE guidelines provide refinement criteria for rodent oncology models. Nat. Protoc. doi: 10.1038/s41596-024-01008-9 38982228

[B1008] Bai L. Zhou D. Li G. Liu J. Chen X. Su J. (2024). Engineering bone/cartilage organoids: Strategy, progress, and application. Bone Research 12, 66. doi: 10.1038/s41413-024-00376-y 39567500 PMC11579019

[B32] Baofeng L. Zhi Y. Bei C. Guolin M. Qingshui Y. Jian L. (2010). Characterization of a rabbit osteoporosis model induced by ovariectomy and glucocorticoid. Acta Orthop. 81, 396–401. doi: 10.3109/17453674.2010.483986. PMID: 20446884 PMC2876847

[B97] Barkaoui A. Tlili B. Vercher-Martínez A. Hambli R. (2016). A multiscale modelling of bone ultrastructure elastic proprieties using finite elements simulation and neural network method. Comput. Methods Programs Biomed. 134, 69–78. doi: 10.1016/j.cmpb.2016.07.005. PMID: 27480733

[B131] Bazyar P. (2024). An overview of developments in computational modeling of bone healing processes. Biomed. J. Sci. Tech. Res. 59, 51524–51534. doi: 10.26717/BJSTR.2024.59.009293

[B236] Bertone P. M. Olevsky L. M. Kathir K. Agnew S. A. Scheideler W. J. Hixon K. R. “ Sintering 3D-printed hydroxyapatite-wollastonite lattices improve bioactivity and mechanical integrity for bone composite scaffolds,” in bioRxiv, 2025.04.06.647463. Preprint. doi: 10.1101/2025.04.06.647463

[B124] Bone‐on‐a‐Chip (2021). Microfluidic Technologies and Microphysiologic Models of Bone Tissue - Mansoorifar ( Advanced Functional Materials - Wiley Online Library). Available online at: https://advanced-onlinelibrary-wiley-com.dartmouth.idm.oclc.org/doi/full/10.1002/adfm.202006796?casa_token=0TLSPY317YMAAAAA%3ADp05qS0WFrdD2TD4uBC9UynLV9Z445XE20kETsisEvMOuAAfWC9kXxLeXHUxLVrkSBfNDt6_ghuFKUc (Accessed March 22, 2026). 10.1002/adfm.202006796 PMC9007546 35422682

[B233] Bone‐on‐a‐Chip (2022). A Microscale 3D Biomimetic Model to Study Bone Regeneration - Galván-Chacón ( Advanced Engineering Materials - Wiley Online Library). doi: 10.1002/adem.202101467

[B160] Braun B. J. Raschke M. J. Schütze K. Pohlemann T. Joeris A. Ernst M. (2025). Prospective first-in-human clinical investigation to evaluate the safety of the fracture monitor T1 in patients with femur fractures treated with a locking compression plate: A study protocol. BMJ Open 15, e102749. doi: 10.1136/bmjopen-2025-102749. PMID: 40744509 PMC12314971

[B60] Caddeo S. Boffito M. Sartori S. (2017). Tissue engineering approaches in the design of healthy and pathological *in vitro* tissue models. Front. Bioeng. Biotechnol. 5. doi: 10.3389/fbioe.2017.00040. PMID: 28798911 PMC5526851

[B238] Cagle A. L. Szulc E. L. Flaggert J. Arias Y. Nikhar A. Tadio D. . (2025). Keratin additive for cellular adhesion in transcutaneous prosthetics. J. Tissue Eng. Regener. Med. 2025, 4337554. doi: 10.1155/term/4337554. PMID: 41476862 PMC12750100

[B116] Cai P. Ding Y. Wang C. Wu J. Hart M. L. Rolauffs B. . (2025). Advancing biomimetic bone scaffolds: from electrospun 2D membranes to functional 3D nanofiber constructs. Biomed. Technol. 11, 100099. doi: 10.1016/j.bmt.2025.100099. PMID: 38826717

[B68] Calejo I. Costa-Almeida R. Gonçalves A. I. Berdecka D. Reis R. L. Gomes M. E. (2018). Bi-directional modulation of cellular interactions in an *in vitro* co-culture model of tendon-to-bone interface. Cell. Prolif 51, e12493. doi: 10.1111/cpr.12493. PMID: 30105786 PMC6528866

[B6] Carbone L. (2021). Estimating mouse and rat use in American laboratories by extrapolation from Animal Welfare Act-regulated species. Sci. Rep. 11, 493. doi: 10.1038/s41598-020-79961-0. PMID: 33436799 PMC7803966

[B120] Chae S. Sun Y. Choi Y. Ha D. Jeon I. Cho D. (2021). 3D cell-printing of tendon-bone interface using tissue-derived extracellular matrix bioinks for chronic rotator cuff repair. Biofabrication 13, 035005. doi: 10.1088/1758-5090/abd159. PMID: 33285539

[B220] Chang M. C. J. Grieder F. B. (2024). The continued importance of animals in biomedical research. Lab. Anim. 53, 295–297. doi: 10.1038/s41684-024-01458-4. PMID: 39402213 PMC11518981

[B36] Changoor A. Suderman R. P. Alshaygy I. Fuhrmann A. Akens M. K. Safir O. . (2022). Irregular porous titanium enhances implant stability and bone ingrowth in an intra-articular ovine model. J. Orthop. Res. 40, 2294–2307. doi: 10.1002/jor.25272. PMID: 35146795

[B76] Cojocaru E. Chiticaru E.-A. Vlăsceanu G. M. Nicolae M.-C. Popescu R.-C. Dobrişan M.-R. . (2026). 3D printing of multicomponent polymeric inks enables hierarchical hybrid architectures for bone tissue regeneration. Mater. Today Adv. 29, 100683. doi: 10.1016/j.mtadv.2025.100683. PMID: 38826717

[B135] Comellas E. Shefelbine S. (2022). The role of computational models in mechanobiology of growing bone. Front. Bioeng. Biotechnol. 10. doi: 10.3389/fbioe.2022.973788. PMID: 36466331 PMC9715592

[B222] Commissioner O of the Animal Welfare . Testing and Research of FDA-Regulated Products ( FDA). Available online at: https://www.fda.gov/consumers/consumer-updates/animal-welfare-testing-and-research-fda-regulated-products.

[B176] Cornell Veterinary Biobank ( Cornell University College of Veterinary Medicine). Available online at: https://www.vet.cornell.edu/departments-centers-and-institutes/cornell-veterinary-biobank (Accessed February 12, 2026).

[B8] Critser R. Locke P. (2024). How should the 3 R’s be revised and why? AMA J. Ethics 26, 724–729. doi: 10.1001/amajethics.2024.724. PMID: 39250945

[B88] D’Amora U. D’Este M. Eglin D. Safari F. Sprecher C. M. Gloria A. . (2018). Collagen density gradient on three-dimensional printed poly(ϵ-caprolactone) scaffolds for interface tissue engineering. J. Tissue Eng. Regener. Med. 12, 321–329. doi: 10.1002/term.2457. PMID: 28486746

[B212] Danridge L. Greer B. Sullivan S. Rutebuka O. DiVincenti L. Wolff A. (2024). Humane endpoints, defined. Lab. Anim. 53, 123–125. doi: 10.1038/s41684-024-01378-3. PMID: 38834808

[B38] Darwiche S. E. Kaczmarek A. Schwarzenberg P. Inglis B. J. Lechmann B. Kronen P. . (2023). Combined electric and magnetic field therapy for bone repair and regeneration: An investigation in a 3-mm and an augmented 17-mm tibia osteotomy model in sheep. J. Orthop. Surg. 18, 454. doi: 10.1186/s13018-023-03910-6. PMID: 37355696 PMC10290367

[B106] Das B. Seesala S. Pal P. Roy T. Roy P. Dhara S. (2021). A vascularized bone-on-a-chip model development via exploring mechanical stimulation for evaluation of fracture healing therapeutics. Vitro Models 1, 73–83. doi: 10.1007/s44164-021-00004-7. PMID: 39872974 PMC11749731

[B173] de Boer J. van Blitterswijk C. Löwik C. (2006). Bioluminescent imaging: Emerging technology for non-invasive imaging of bone tissue engineering. Biomaterials 27, 1851–1858. doi: 10.1016/j.biomaterials.2005.09.034. PMID: 16242768

[B210] De Vleeschauwer S. I. van de Ven M. Oudin A. Debusschere K. Connor K. Byrne A. T. . (2024). OBSERVE: guidelines for the refinement of rodent cancer models. Nat. Protoc. 19, 2571–2596. doi: 10.1038/s41596-024-00998-w. PMID: 38992214

[B219] Diederich K. Schmitt K. Schwedhelm P. Bert B. Heinl C. (2022). A guide to open science practices for animal research. PLoS Biol. 20, e3001810. doi: 10.1371/journal.pbio.3001810. PMID: 36108043 PMC9514607

[B123] Diniz P. Grimm B. Garcia F. Fayad J. Ley C. Mouton C. . (2025). Digital twin systems for musculoskeletal applications: a current concepts review. Knee Surg. Sports Traumatol. Arthrosc Off. J. ESSKA 33. doi: 10.1002/ksa.12627. PMID: 39989345

[B104] Duval K. Grover H. Han L.-H. Mou Y. Pegoraro A. F. Fredberg J. . (2017). Modeling physiological events in 2D vs. 3D cell culture. Physiology 32, 266–277. doi: 10.1152/physiol.00036.2016. PMID: 28615311 PMC5545611

[B195] Ehx G. Ritacco C. Baron F. (2024). Pathophysiology and preclinical relevance of experimental graft-versus-host disease in humanized mice. biomark. Res. 12, 139. doi: 10.1186/s40364-024-00684-9. PMID: 39543777 PMC11566168

[B169] Einafshar M. Najafidoust M. Bastami F. Massaad E. Hashemi A. Kiapour A. Nondestructive acoustic modal analysis for assessing bone screw stability: An ex vivo animal study. doi: 10.1002/jor.25959 39191523

[B47] Eldeeb D. W. Hommos A. M. Taalab M. R. Abd El Rehim S. S. (2023). Immuno-histologic and histomorphometric evaluation of Angelica sinensis adjunctive to ß-tricalcium phosphate in critical-sized class II furcation defects in dogs. BDJ Open 9, 23. doi: 10.1038/s41405-023-00150-y. PMID: 37353505 PMC10290112

[B69] Engler A. J. Sen S. Sweeney H. L. Discher D. E. (2006). Matrix elasticity directs stem cell lineage specification. Cell. 126, 677–689. doi: 10.1016/j.cell.2006.06.044. PMID: 16923388

[B223] Ethical use of animals in medicine testing ( European Medicines Agency (EMA). Available online at: https://www.ema.europa.eu/en/human-regulatory-overview/research-development/ethical-use-animals-medicine-testing (Accessed January 5, 2026).

[B209] Falcon J. M. Karchner J. P. Henning E. A. Mauck R. L. Pleshko N. (2019). Ethics of using animal models as predictors of human response in tissue engineering. Ethics Biol. Eng. Med. 10, 37–49. doi: 10.1615/EthicsBiologyEngMed.2020033718. PMID: 38770223 PMC11103864

[B96] Fielder M. Nair A. K. (2024). Predicting ultrasound wave stimulated bone growth in bioinspired scaffolds using machine learning. J. Mech. Behav. Biomed. Mater. 159, 106684. doi: 10.1016/j.jmbbm.2024.106684. PMID: 39178821

[B10] Flores M. J. Brown K. E. O’Marr J. M. Adejuyigbe B. Rodarte P. Gomez-Alvarado F. . (2024). The economic impact of infection and/or nonunion on long-bone shaft fractures: a systematic review. OTA Int. 7, e337. doi: 10.1097/OI9.0000000000000337. PMID: 38863461 PMC11164001

[B186] Frühwein H. Paul N. W. (2025). Lost in translation?” Animal research in the era of precision medicine. J. Transl. Med. 23, 152. doi: 10.1186/s12967-025-06084-3. PMID: 39905446 PMC11796152

[B100] Fu R. Chen Z. Tian H. Hu J. Bu F. Zheng P. . (2025). A review on the applications of machine learning in biomaterials, biomechanics, and biomanufacturing for tissue engineering. Smart Mater. Med. 6, 171–204. doi: 10.1016/j.smaim.2025.06.003. PMID: 38826717

[B49] Fukuba S. Akizuki T. Matsuura T. Okada M. Nohara K. Hoshi S. . (2021). Effects of combined use of recombinant human fibroblast growth factor-2 and β-tricalcium phosphate on ridge preservation in dehiscence bone defects after tooth extraction: A split-mouth study in dogs. J. Periodontal Res. 56, 298–305. doi: 10.1111/jre.12818. PMID: 33314140

[B20] Gao H. Huang J. Wei Q. He C. (2023). Advances in animal models for studying bone fracture healing. Bioengineering 10, 201. doi: 10.3390/bioengineering10020201. PMID: 36829695 PMC9952559

[B129] García-Aznar J. Nasello G. Hervas-Raluy S. Pérez M. Gómez-Benito M. (2021). Multiscale modeling of bone tissue mechanobiology. Bone 151, 116032. doi: 10.1016/j.bone.2021.116032. PMID: 34118446

[B189] Gelles K. Butylina M. Pietschmann P. (2025). Animal models for age-related osteoporosis. Gerontology 71, 755–772. doi: 10.1159/000546107. PMID: 40418907 PMC12349892

[B9] Ghanem W. Ezzeddine H. Saad R. Kiwan E. Dahdouh R. Fakih O. . State of the nonunion: A review of the latest literature. Orthop. Rev. 17, 129085. doi: 10.52965/001c.129085. PMID: 39925644 PMC11807701

[B39] Ghosh Y. A. Xin H. Al Maruf D. S. A. Cheng K. Wise I. Burrows C. . (2024). Novel sheep model to assess critical-sized bone regeneration with periosteum for *in vivo* bioreactors. Tissue Eng. Part. C. Methods 30, 159–169. doi: 10.1089/ten.tec.2023.0345. PMID: 38368556

[B12] Gillman C. E. Jayasuriya A. C. (2021). FDA-approved bone grafts and bone graft substitute devices in bone regeneration. Mater. Sci. Eng. C. Mater. Biol. Appl. 130, 112466. doi: 10.1016/j.msec.2021.112466. PMID: 34702541 PMC8555702

[B187] Gliksten L. Yip P. K. (2023). Current spinal cord injury animal models are too simplistic for clinical translation. J. Exp. Neurol. 4, 6–10. doi: 10.33696/Neurol.4.068

[B213] Göpel T. Burggren W. W. (2022). Insufficient reporting of experimental variables as a cause for nonreproducibility in animal physiology? A case study. Am. J. Physiol. - Regul. Integr. Comp. Physiol. 323, R363–R374. doi: 10.1152/ajpregu.00026.2022. PMID: 35816721 PMC9467468

[B1] Grimm H. Biller-Andorno N. Buch T. Dahlhoff M. Davies G. Cederroth C. R. . (2023). Advancing the 3Rs: innovation, implementation, ethics and society. Front. Vet. Sci. 10. doi: 10.3389/fvets.2023.1185706. PMID: 37396988 PMC10310538

[B30] Guo J. L. Kim Y. S. Orchard E. A. van den Beucken J. J. J. P. Jansen J. A. Wong M. E. . (2020). A rabbit femoral condyle defect model for assessment of osteochondral tissue regeneration. Tissue Eng. Part. C. Methods 26, 554–564. doi: 10.1089/ten.tec.2020.0261. PMID: 33050806 PMC7698983

[B163] Haberthür D. Khoma O.-Z. Hoessly T. Zoni E. Julio M. K. Ryan S. D. . (2025). MicroCT-based vascular imaging in bone and peri-implant tissues. Tomogr. Mater. Struct. 9, 100074. doi: 10.1016/j.tmater.2025.100074. PMID: 38826717

[B16] Hadjiargyrou M. Hildreth B. E. Ko F. Sankar U. Yang T. (2026). The scientific case for animal models: A perspective from musculoskeletal researchers. FASEB Bioadv. 8, e70090. doi: 10.1096/fba.2025-00313. PMID: 41669563 PMC12885169

[B103] Handelman G. Kok H. Chandra R. Razavi A. Lee M. Asadi H. (2018). eDoctor: machine learning and the future of medicine. J. Intern. Med. 284, 603–619. doi: 10.1111/joim.12822. PMID: 30102808

[B109] Hao Z. Xu Z. Wang X. Wang Y. Li H. Chen T. . (2021). Biophysical stimuli as the fourth pillar of bone tissue engineering. Front. Cell Dev. Biol. 9. doi: 10.3389/fcell.2021.790050. PMID: 34858997 PMC8630705

[B35] Harrison K. D. Hiebert B. D. Panahifar A. Andronowski J. M. Ashique A. M. King G. A. . (2020). Cortical bone porosity in rabbit models of osteoporosis. J. Bone Miner. Res. Off. J. Am. Soc Bone Miner. Res. 35, 2211–2228. doi: 10.1002/jbmr.4124. PMID: 32614975 PMC7702175

[B75] Hassan S. N. Abdelkhalek M. A. Gamal A. Y. El-Kemary M. A. Abdel Gaber S. A. (2026). Simvastatin loaded marine collagen-silk fibroin electrospun nanofiber as a bioactive guided tissue membrane for regenerative and anti-inflammatory therapy. J. Drug Delivery Sci. Technol. 117, 108062. doi: 10.1016/j.jddst.2026.108062. PMID: 38826717

[B193] Hawkins P. Dooley J. Rodda J. Gilbert C. (2025). Refining bone marrow ablation and reconstitution in mice. Immunol. Cell Biol. 103, 293–306. doi: 10.1111/imcb.12847. PMID: 39788714 PMC11884310

[B138] Hedayatzadeh Razavi A. Nafisi N. Ghiasi M. S. Oftadeh R. Hanna P. Lechtig A. . (2024). A computational model that integrates unrestricted callus growth, mechanobiology, and angiogenesis can predict bone healing in rodents. Sci. Rep. 14, 29826. doi: 10.1038/s41598-024-80502-2. PMID: 39616231 PMC11608358

[B19] Hixon K. R. Miller A. N. (2022). Animal models of impaired long bone healing and tissue engineering- and cell-based *in vivo* interventions. J. Orthop. Res. 40, 767–778. doi: 10.1002/jor.25277. PMID: 35072292

[B46] Ho K. N. Salamanca E. Chang K.-C. Shih T.-C. Chan J. Huang H.-M. . (2016). A novel HA/β-TCP-collagen composite enhanced new bone formation for dental extraction socket preservation in beagle dogs. Materials 9, 191. doi: 10.3390/ma9030191. PMID: 28773317 PMC5456700

[B42] Hojo S. Bamba N. Kojima K. Kodama T. (2020). Examination of β-TCP/collagen composite in bone defects without periosteum in dogs: A histological and cast model evaluation. Odontology 108, 578–587. doi: 10.1007/s10266-020-00506-y. PMID: 32162098

[B196] Holler E. Greinix H. Zeiser R. “ Acute graft-versus-host disease,” in The EBMT Handbook: Hematopoietic Cell Transplantation and Cellular Therapies. Available online at: http://www.ncbi.nlm.nih.gov/books/NBK608233/.

[B228] Hong Y. Li R. Sheng S. Zhou F. Bai L. Su J. (2025). Bone organoid construction and evolution. J. Orthop. Transl. 53, 260–273. doi: 10.1016/j.jot.2025.06.011. PMID: 40687551 PMC12270736

[B231] Hu Y. Zhang H. Wang S. Cao L. Zhou F. Jing Y. . (2023). Bone/cartilage organoid on-chip: construction strategy and application. Bioact. Mater. 25, 29–41. doi: 10.1016/j.bioactmat.2023.01.016. PMID: 37056252 PMC10087111

[B90] Huang M. S. Christakopoulos F. Roth J. G. Heilshorn S. C. (2025). Organoid bioprinting: from cells to functional tissues. Nat. Rev. Bioeng. 3, 126–142. doi: 10.1038/s44222-024-00268-0. PMID: 37880705

[B154] Huang M. Dissanayaka W. L. Yiu C. K. Y. (2025). Artificial intelligence driven innovation: Advancing mesenchymal stem cell therapies and intelligent biomaterials for regenerative medicine. Bioengineering 12, 1302. doi: 10.3390/bioengineering12121302. PMID: 41463599 PMC12729526

[B229] Huang S. Wu Y. Zhao H. Song K. Liu Y. Mao J. . Advancements in bone organoids: perspectives on construction methodologies and application strategies. J. Adv. Res. doi: 10.1016/j.jare.2025.06.011. PMID: 40513657 PMC12957805

[B11] Huang E. E. Zhang N. Shen H. . (2022). Novel techniques and future perspective for investigating critical-size bone defects. Bioengineering 9, 171. doi: 10.3390/bioengineering9040171. PMID: 35447731 PMC9027954

[B54] Hwang S.-C. Hwang D. S. Kim H. Y. Kim M. J. Kang Y.-H. Byun S.-H. . (2019). Development of bone regeneration strategies using human periosteum-derived osteoblasts and oxygen-releasing microparticles in mandibular osteomyelitis model of miniature pig. J. Biomed. Mater. Res. A. 107, 2183–2194. doi: 10.1002/jbm.a.36728. PMID: 31116505

[B201] Hyndman T. H. Bowden R. S. Woodward A. P. Pang D. S. J. Hampton J. O. (2024). Uncontrolled pain: a call for better study design. Front. Vet. Sci. 11. doi: 10.3389/fvets.2024.1328098. PMID: 38420206 PMC10899387

[B200] Iafrate L. Benedetti M. C. Donsante S. Rosa A. Corsi A. Oreffo R. O. C. . (2022). Modelling skeletal pain harnessing tissue engineering. Vitro Models 1, 289–307. doi: 10.1007/s44164-022-00028-7. PMID: 36567849 PMC9766883

[B144] “ In silico bone mechanobiology: modeling a multifaceted biological system - Giorgi,” in WIREs Systems Biology and Medicine ( Wiley Online Library) (2016). Available online at: https://wires-onlinelibrary-wiley-com.dartmouth.idm.oclc.org/doi/full/10.1002/wsbm.1356 (Accessed January 27, 2026). 10.1002/wsbm.1356 PMC5082538 27600060

[B118] Iordachescu A. Hughes E. Joseph S. Hill E. Grover L. Metcalfe A. (2021). Trabecular bone organoids: a micron-scale ‘humanised’ prototype designed to study the effects of microgravity and degeneration. NPJ Microgravity 7, 17. doi: 10.1038/s41526-021-00146-8. PMID: 34021163 PMC8140135

[B102] Javaid S. Gorji H. Soulami K. Kaabouch N. (2023). Identification and ranking biomaterials for bone scaffolds using machine learning and PROMETHEE. Res. Biomed. Eng. 39, 129–138. doi: 10.1007/s42600-022-00257-5. PMID: 30311153

[B72] Jenkins T. L. Little D. (2019). Synthetic scaffolds for musculoskeletal tissue engineering: cellular responses to fiber parameters. NPJ Regener. Med. 4, 15. doi: 10.1038/s41536-019-0076-5. PMID: 31263573 PMC6597555

[B105] Jensen C. Teng Y. (2020). Is it time to start transitioning from 2D to 3D cell culture? Front. Mol. Biosci. 7. doi: 10.3389/fmolb.2020.00033. PMID: 32211418 PMC7067892

[B159] Jeznach O. Tabakoglu S. Zaszczyńska A. Sajkiewicz P. (2024). Review on machine learning application in tissue engineering: What has been done so far? Application areas, challenges, and perspectives. J. Mater. Sci. 59, 21222–21250. doi: 10.1007/s10853-024-10449-2. PMID: 30311153

[B179] Jiang S. Knapstein P. Donat A. Tsitsilonis S. Keller J. (2021). An optimized protocol for a standardized, femoral osteotomy model to study fracture healing in mice. STAR Protoc. 2, 100798. doi: 10.1016/j.xpro.2021.100798. PMID: 34527955 PMC8433248

[B98] Jung G. S. Buehler M. J. (2017). Multiscale modeling of muscular-skeletal systems. Annu. Rev. Biomed. Eng. 19, 435–457. doi: 10.1146/annurev-bioeng-071516-044555. PMID: 28460181

[B148] Kameo Y. Miya Y. Hayashi M. Nakashima T. Adachi T. (2020). In silico experiments of bone remodeling explore metabolic diseases and their drug treatment. Sci. Adv. 6, eaax0938. doi: 10.1126/sciadv.aax0938. PMID: 32181336 PMC7060067

[B235] Kang X. Zhang X. B. Gao X. D. Hao D. J. Li T. Xu Z. W. (2022). Bioprinting for bone tissue engineering. Front. Bioeng. Biotechnol. 10. doi: 10.3389/fbioe.2022.1036375. PMID: 36507261 PMC9732272

[B114] Kapałczyńska M. Kolenda T. Przybyła W. Zajączkowska M. Teresiak A. Filas V. . (2016). 2D and 3D cell cultures – a comparison of different types of cancer cell cultures. Arch. Med. Sci. 14 (4), 910–919. doi: 10.5114/aoms.2016.63743. PMID: 30002710 PMC6040128

[B224] Kiani A. K. Pheby D. Henehan G. Brown R. Bertelli M. (2022). Ethical considerations regarding animal experimentation. J. Prev. Med. Hyg. 63, E255–E266. doi: 10.15167/2421-4248/jpmh2022.63.2S3.2768. PMID: 36479489 PMC9710398

[B165] Kim Y. Brodt M. D. Tang S. Y. Silva M. J. (2021). MicroCT for scanning and analysis of mouse bones. Methods Mol. Biol. Clifton NJ 2230, 169–198. doi: 10.1007/978-1-0716-1028-2_11. PMID: 33197015 PMC8409170

[B44] Kim D. W. Lee D. Ryu J. Kook M. S. Park H. J. Jung S. (2025). Evaluation of hydroxyapatite–β-tricalcium phosphate collagen composites for socket preservation in a canine model. J. Funct. Biomater. 16, 286. doi: 10.3390/jfb16080286. PMID: 40863306 PMC12387299

[B1009] Kim G. Jeon J. H. Park K. Kim S. W. Kim D. H. Lee S. (2022). High throughput screening of mesenchymal stem cell lines using deep learning. Scientific Reports 12, (1), 17507. doi: 10.1038/s41598-022-21653-y 36266301 PMC9584889

[B18] Koh N. Y. Y. Miszkiewicz J. J. Fac M. L. Wee N. K. Y. Sims N. A. (2024). Preclinical rodent models for human bone disease, including a focus on cortical bone. Endocr. Rev. 45, 493–520. doi: 10.1210/endrev/bnae004. PMID: 38315213 PMC11244217

[B146] Koller W. Kranzl A. Mindler G. Baca A. Kainz H. (2024). Advanced multi-scale mechanobiological simulations enable the distinction between healthy and pathological bone growth patterns. Curr. Issues Sport Sci. CISS 9, 007–007. doi: 10.36950/2024.4ciss007

[B151] Kolomenskaya E. Butova V. Poltavskiy A. Soldatov A. Butakova M. (2024). Application of artificial intelligence at all stages of bone tissue engineering. Biomedicines 12, 76. doi: 10.3390/biomedicines12010076. PMID: 38255183 PMC10813365

[B136] Kuba S. Simpson M. Buenzli P. (2026). Mechanistic interpretation of biological tissue growth experiments with a computational model. bioRxiv, 2026.03.12.711199. doi: 10.64898/2026.03.12.711199. Preprint.

[B128] Kumar R. Gowda C. Paladugu P. Younis Y. Marla K. Gowda N. . (2025). Computational Modelling of Bone Remodelling: Integration of Finite Element Analysis, Machine Learning, and Mechanobiological Principles. Mach Learn. 8, (1).

[B56] Lad S. E. (2023). Absence of secondary osteons in femora of aged rats: Implications of lifespan on Haversian remodeling in mammals. J. Morphol. 284, e21600. doi: 10.1002/jmor.21600. PMID: 37313764

[B177] Lang A. (2022). Implementation of the 3R principle in musculoskeletal research – Refinement measures and *in vitro* replacement methods. doi: 10.17169/refubium-37005

[B125] Lee S. Kim Y. G. Jung H.-I. Lim J. S. Nam K. C. Choi H. S. . (2024). Bone-on-a-chip simulating bone metastasis in osteoporosis. Biofabrication 16. doi: 10.1088/1758-5090/ad6cf9. PMID: 39116896

[B82] Lee J. Lee S. Huh S. J. Kang B. J. Shin H. (2022). Directed regeneration of osteochondral tissue by hierarchical assembly of spatially organized composite spheroids. Adv. Sci. 9, 2103525. doi: 10.1002/advs.202103525. PMID: 34806336 PMC8787388

[B27] Lei W. Wu Y. Yuan H. He P. Wu J. (2025). Establishing rabbit critical-size bone defects to evaluate the bone-regeneration potential of porous calcium phosphate ceramics. Front. Bioeng. Biotechnol. 12. doi: 10.3389/fbioe.2024.1524133. PMID: 39944476 PMC11813868

[B232] Lei Y. Zhang Y. Zhou L. Li Z. A. (2025). Organoids and organs-on-chips for accelerating R&D and clinical translation in orthopaedics: emerging opportunities and regulatory pathways. J. Orthop. Transl., 101021. doi: 10.1016/j.jot.2025.10.013. PMID: 41836578 PMC12988525

[B113] Lelièvre S. Kwok T. Chittiboyina S. (2017). Architecture in 3D cell culture: an essential feature for *in vitro* toxicology. Toxicol. Vitro 45, 287–295. doi: 10.1016/j.tiv.2017.03.012. PMID: 28366709 PMC5623165

[B108] Leppik L. Oliveira K. Bhavsar M. Barker J. (2020). Electrical stimulation in bone tissue engineering treatments. Eur. J. Trauma Emerg. Surg. 46, 231–244. doi: 10.1007/s00068-020-01324-1. PMID: 32078704 PMC7113220

[B95] Leung C. M. de Haan P. Ronaldson-Bouchard K. Kim G.-A. Ko J. Rho H. S. . (2022). A guide to the organ-on-a-chip. Nat. Rev. Methods Primer 2, 33. doi: 10.1038/s43586-022-00118-6. PMID: 37880705

[B52] Li Y. Chen S. K. Li L. Qin L. Wang X. L. Lai Y. X. (2015). Bone defect animal models for testing efficacy of bone substitute biomaterials. J. Orthop. Transl. 3, 95–104. doi: 10.1016/j.jot.2015.05.002. PMID: 30035046 PMC5982383

[B74] Li Y. Kilian K. A. (2015). Bridging the gap: from 2D cell culture to 3D microengineered extracellular matrices. Adv. Healthc Mater. 4, 2780–2796. doi: 10.1002/adhm.201500427. PMID: 26592366 PMC4780579

[B127] Li Z. Lin Z. Liu S. Yagi H. Zhang X. Yocum L. . (2022). Human mesenchymal stem cell-derived miniature joint system for disease modeling and drug testing. Adv. Sci. 9, 2105909. doi: 10.1002/advs.202105909. PMID: 35436042 PMC9313499

[B21] Li J. Sarosi I. Yan X. Q. Morony S. Capparelli C. Tan H. L. . (2000). RANK is the intrinsic hematopoietic cell surface receptor that controls osteoclastogenesis and regulation of bone mass and calcium metabolism. Proc. Natl. Acad. Sci. U.S.A. 97, 1566–1571. doi: 10.1073/pnas.97.4.1566. PMID: 10677500 PMC26475

[B155] Li C. Zhong Z. Zhang N. Yuan S. Fang R. Ge F. . (2026). Artificial intelligence in bone biology: Transforming basic research into advanced therapeutics. Innov. Life. 4, 100186–100113. doi: 10.59717/j.xinn-life.2026.100186

[B180] Lin Y. Jiang Z. Yang J. Wang M. Wang H. Zhang X. . (2024a). Development of a standardized and reproducible murine femoral distraction osteogenesis model. J. Orthop. Transl. 49, 74–81. doi: 10.1016/j.jot.2024.08.001. PMID: 39430129 PMC11488447

[B182] Lin Y. Jiang Z. Yang J. Wang M. Wang H. Zhang X. . (2024b). Development of a standardized and reproducible murine femoral distraction osteogenesis model. J. Orthop. Transl. 49, 74–81. doi: 10.1016/j.jot.2024.08.001. PMID: 39430129 PMC11488447

[B61] Lipreri M. V. Di Pompo G. Boanini E. Graziani G. Sassoni E. Baldini N. . (2023). Bone on-a-chip: a 3D dendritic network in a screening platform for osteocyte-targeted drugs. Biofabrication 15, 045019. doi: 10.1088/1758-5090/acee23. PMID: 37552982

[B111] Liu J. Wang X. Wu Z. Hui Z. Zhang J. Zhang J. . (2026). Dynamic stimulation of piezoelectric scaffolds enhances osteogenesis-related biological responses via electro-mechanical sensitive channels and cytoskeletal remodeling. Biomaterials 329, 123938. doi: 10.1016/j.biomaterials.2025.123938. PMID: 41456503

[B79] Liu Y. Zhou Z. Lu G. Zhang X. Shi D. Tong L. . (2025). Musculoskeletal organoids: An emerging toolkit for establishing personalized models of musculoskeletal disorders and developing regenerative therapies. Acta Biomater. 200, 158–186. doi: 10.1016/j.actbio.2025.05.037. PMID: 40381929

[B171] Logeart-Avramoglou D. Oudina K. Bourguignon M. Delpierre L. Nicola M.-A. Bensidhoum M. . (2010). *In vitro* and *in vivo* bioluminescent quantification of viable stem cells in engineered constructs. Tissue Eng. Part. C. Methods 16, 447–458. doi: 10.1089/ten.tec.2009.0004. PMID: 19624260

[B137] López-Vaca R. Narváez-Tovar C. A. Das R. de Boer G. Ramtani S. Boucetta A. . (2025). Mechanobiological model of endochondral ossification and trabecular bone modeling. Int. J. Numer. Methods Biomed. Eng. 41, e70024. doi: 10.1002/cnm.70024. PMID: 40084415

[B41] Lu H. Zhou Y. Ma Y. Xiao L. Ji W. Zhang Y. . (2021). Current application of beta-tricalcium phosphate in bone repair and its mechanism to regulate osteogenesis. Front. Mater. 8. doi: 10.3389/fmats.2021.698915

[B142] Madden J. Enoch S. Paini A. Cronin M. (2020). A review of in silico tools as alternatives to animal testing: principles, resources and applications. Altern. Lab. Anim. 48, 146–172. doi: 10.1177/0261192920965977. PMID: 33119417

[B199] Magnusdottir R. Gohin S. ter Heegde F. Hopkinson M. McNally I. F. Fisher A. . (2021). Fracture-induced pain-like behaviours in a femoral fracture mouse model. Osteoporos Int. 32, 2347–2359. doi: 10.1007/s00198-021-05991-7. PMID: 34080043 PMC8563675

[B1011] Mai M. Luo S. Fasciano S. Oluwole T. E. Ortiz J. Pang Y. . (2023). Morphology-based deep learning approach for predicting adipogenic and osteogenic differentiation of human mesenchymal stem cells (hMSCs). Frontiers in Cell and Developmental Biology 11, 1329840. doi: 10.3389/fcell.2023.1329840 38099293 PMC10720363

[B208] Management of Laboratory Animals Exotic and Laboratory Animals. Merck Veterinary Manual. Available online at: https://www.merckvetmanual.com/exotic-and-laboratory-animals/laboratory-animals/management-of-laboratory-animals (Accessed February 12, 2026).

[B234] Maresca J. A. DeMel D. C. Wagner G. A. Haase C. Geibel J. P. (2023). Three-dimensional bioprinting applications for bone tissue engineering. Cells 12, 1230. doi: 10.3390/cells12091230. PMID: 37174630 PMC10177443

[B85] Marrella A. Aiello M. Quarto R. Scaglione S. (2016). Chemical and morphological gradient scaffolds to mimic hierarchically complex tissues: From theoretical modeling to their fabrication. Biotechnol. Bioeng. 113, 2286–2297. doi: 10.1002/bit.25994. PMID: 27093435

[B185] Marshall L. J. Bailey J. Cassotta M. Herrmann K. Pistollato F. (2023). Poor translatability of biomedical research using animals — A narrative review. Altern. Lab. Anim. 51, 102–135. doi: 10.1177/02611929231157756. PMID: 36883244

[B147] Marsilio L. Barrios S. Maksimovic S. Maccarini A. Serafini E. Grimaldi M. . (2025). In silico digital twins of bone metastasis enable investigation of tumor progression and therapy response. Cancer Res. 85, 4269–4284. doi: 10.1158/0008-5472.CAN-25-0088. PMID: 40965325

[B77] Martin I. Wendt D. Heberer M. (2004). The role of bioreactors in tissue engineering. Trends Biotechnol. 22, 80–86. doi: 10.1016/j.tibtech.2003.12.001. PMID: 14757042

[B203] McVeigh L. G. Perugini A. J. Fehrenbacher J. C. White F. A. Kacena M. A. (2020). Assessment, quantification, and management of fracture pain: from animals to the clinic. Curr. Osteoporos Rep. 18, 460–470. doi: 10.1007/s11914-020-00617-z. PMID: 32827293 PMC7541703

[B63] Miki Y. Ono K. Hata S. Suzuki T. Kumamoto H. Sasano H. (2012). The advantages of co-culture over mono cell culture in simulating *in vivo* environment. J. Steroid Biochem. Mol. Biol. 131, 68–75. doi: 10.1016/j.jsbmb.2011.12.004. PMID: 22265957

[B40] Mosaddad S. A. Hussain A. Tebyaniyan H. (2024). Exploring the use of animal models in craniofacial regenerative medicine: A narrative review. Tissue Eng. Part. B. Rev. 30, 29–59. doi: 10.1089/ten.teb.2023.0038. PMID: 37432898

[B145] Multiscale modeling of bone tissue mechanobiology. ScienceDirect. doi: 10.1016/j.bone.2021.116032 34118446

[B166] Namiranian B. Doi K. Alenezi S. Shah S. B. Jerban S. Chang E. Y. (2025). Bone evaluation with micro finite element analysis in animal models. Tomography 11, 101. doi: 10.3390/tomography11090101. PMID: 41003484 PMC12473714

[B115] Nelson C. Bissell M. (2006). Of extracellular matrix, scaffolds, and signaling: tissue architecture regulates development, homeostasis, and cancer. Annu. Rev. Cell Dev. Biol. 22, 287–309. doi: 10.1146/annurev.cellbio.22.010305.104315. PMID: 16824016 PMC2933192

[B141] Notermans T. Isaksson H. (2023). Predicting the formation of different tissue types during Achilles tendon healing using mechanoregulated and oxygen-regulated frameworks. Biomech. Model. Mechanobiol. 22, 655–667. doi: 10.1007/s10237-022-01672-4. PMID: 36542228 PMC10097799

[B197] Nowak A. Marlow R. Ryan K. Lapointe J.-M. Sutton D. Sharpe A. . (2025). A comprehensive welfare scoring system for graft versus host disease clinical assessment in humanised mouse models used for pharmaceutical research. Front. Immunol. 16. doi: 10.3389/fimmu.2025.1617528. PMID: 40607426 PMC12213481

[B140] Ntousi O. Roumpi M. Siogkas P. K. Polyzos D. Kakkos I. Matsopoulos G. K. . (2025). Advances in computational modeling of scaffolds for bone tissue engineering: a narrative review of the current approaches and challenges. Biomechanics 5. doi: 10.3390/biomechanics5040076. PMID: 30654563

[B34] Oki Y. Doi K. Kobatake R. Makihara Y. Morita K. Kubo T. . (2022). Histological and histomorphometric aspects of continual intermittent parathyroid hormone administration on osseointegration in osteoporosis rabbit model. PLoS One 17, e0269040. doi: 10.1371/journal.pone.0269040. PMID: 35675357 PMC9176794

[B167] Olăreț E. Stancu I. C. Iovu H. Serafim A. (2021). Computed tomography as a characterization tool for engineered scaffolds with biomedical applications. Materials 14, 6763. doi: 10.3390/ma14226763. PMID: 34832165 PMC8619049

[B237] Olevsky L. M. Anup A. Jacques M. Keokominh N. Holmgren E. P. Hixon K. R. (2023). Direct integration of 3D printing and cryogel scaffolds for bone tissue engineering. Bioengineering 10. doi: 10.3390/bioengineering10080889. PMID: 37627774 PMC10451777

[B152] Paek K. Kim S. Tak S. Kim M. K. Park J. Chung S. . (2023). A high-throughput biomimetic bone-on-a-chip platform with artificial intelligence-assisted image analysis for osteoporosis drug testing. Bioeng. Transl. Med. 8, e10313. doi: 10.1002/btm2.10313. PMID: 36684077 PMC9842054

[B206] Park J. Fertala A. Tomlinson R. E. (2019). Naproxen impairs load-induced bone formation, reduces bone toughness, and diminishes woven bone formation following stress fracture in mice. Bone 124, 22–32. doi: 10.1016/j.bone.2019.04.009. PMID: 30998998

[B217] Percie du Sert N. Hurst V. Ahluwalia A. Alam S. Avey M. T. Baker M. . (2020). The ARRIVE guidelines 2.0: updated guidelines for reporting animal research*. J. Cereb. Blood Flow Metab. 40, 1769–1777. doi: 10.1177/0271678X20943823. PMID: 32663096 PMC7430098

[B143] Perier-Metz C. Cipitria A. Hutmacher D. Duda G. Checa S. (2022a). An in silico model predicts the impact of scaffold design in large bone defect regeneration. Acta Biomater. 145, 329–341. doi: 10.1016/j.actbio.2022.04.008. PMID: 35417799

[B134] Perier-Metz C. Duda G. Checa S. (2022b). A mechanobiological computer optimization framework to design scaffolds to enhance bone regeneration. Front. Bioeng. Biotechnol. 10. doi: 10.3389/fbioe.2022.980727. PMID: 36159680 PMC9490117

[B71] Persson K. M. Whitman M. A. Fischbach C. (2026). Engineered bone matrix models for understanding breast cancer skeletal metastasis. Cancer Metastasis Rev. 45, 4. doi: 10.1007/s10555-025-10310-1. PMID: 41579220 PMC12831698

[B70] Ragni E. de Girolamo L. Kouroupis D. (2025). Harnessing the mechanical microenvironment to optimize mesenchymal stem/stromal cells (MSCs) extracellular vesicle therapeutics. Ann. Transl. Med. 13, 67. doi: 10.21037/atm-25-145. PMID: 41502443 PMC12771047

[B168] Ramirez S. P. Hernandez I. Balcorta H. V. Kumar P. Kumar V. Poon W. . (2024). Microcomputed tomography for the microstructure evaluation of 3D bioprinted scaffolds. ACS Appl. Bio Mater. 7, 7799–7808. doi: 10.1021/acsabm.3c00621. PMID: 37871142

[B107] Rauh J. Milan F. Günther K. Stiehler M. (2011). Bioreactor systems for bone tissue engineering. Tissue Eng. Part. B. Rev. 17, 263–280. doi: 10.1089/ten.teb.2010.0612. PMID: 21495897

[B4] Research Facility Annual Report Summary (2024). ( Fiscal). Available online at: https://www.aphis.usda.gov/sites/default/files/fy2024-research-animal-use-summary.pdf (Accessed January 19, 2026).

[B221] Ribitsch I. Baptista P. M. Lange-Consiglio A. Melotti L. Patruno M. Jenner F. . (2020). Large animal models in regenerative medicine and tissue engineering: to do or not to do. Front. Bioeng. Biotechnol. 8. doi: 10.3389/fbioe.2020.00972. PMID: 32903631 PMC7438731

[B194] Rios C. I. Hollingsworth B. A. DiCarlo A. L. Esker J. E. Satyamitra M. M. Silverman T. A. . (2022). Animal care in radiation medical countermeasures studies. Radiat. Res. 198, 514–535. doi: 10.1667/RADE-21-00211.1. PMID: 36001810 PMC9743977

[B14] Rodriguez-Palomo A. Østergaard M. Birkedal H. (2024). Bone hierarchical structure: Heterogeneity and uniformity. Adv. Funct. Mater. 34, 2307026. doi: 10.1002/adfm.202307026. PMID: 41531421

[B139] Rowe J. Shen S. de Alcântara A. C. S. Skaf M. S. Dini D. Harrison N. M. . (2025). Integrating computational and experimental advances in bone multiscale mechanics. Prog. Mater Sci. 153, 101474. doi: 10.1016/j.pmatsci.2025.101474. PMID: 38826717

[B150] Sabet F. Raeisi Najafi A. Hamed E. Jasiuk I. (2016). Modelling of bone fracture and strength at different length scales: a review. Interface Focus 6, 20150055. doi: 10.1098/rsfs.2015.0055. PMID: 26855749 PMC4686238

[B33] Sadat-Ali M. Al-Dakheel D. A. Al-Turki H. A. Acharya S. (2021). Efficacy of autologous bone marrow derived Mesenchymal stem cells (MSCs), osteoblasts and osteoblasts derived exosome in the reversal of ovariectomy (OVX) induced osteoporosis in rabbit model. Am. J. Transl. Res. 13, 6175–6181. Available online at: https://pmc.ncbi.nlm.nih.gov/articles/PMC8290779/. 34306356 PMC8290779

[B29] Sadek A. A. Abd-Elkareem M. Abdelhamid H. N. Moustafa S. Hussein K. (2023). Repair of critical-sized bone defects in rabbit femurs using graphitic carbon nitride (g-C3N4) and graphene oxide (GO) nanomaterials. Sci. Rep. 13, 5404. doi: 10.1038/s41598-023-32487-7. PMID: 37012344 PMC10070441

[B172] Sadikot R. T. Blackwell T. S. (2005). Bioluminescence imaging. Proc. Am. Thorac. Soc 2, 537–540. doi: 10.1513/pats.200507-067DS. PMID: 16352761 PMC2713342

[B184] Saunders W. B. Dejardin L. M. Soltys-Niemann E. V. Kaulfus C. N. Eichelberger B. M. Dobson L. K. . (2022). Angle-stable interlocking nailing in a canine critical-sized femoral defect model for bone regeneration studies: In pursuit of the principle of the 3R’s. Front. Bioeng. Biotechnol. 10. doi: 10.3389/fbioe.2022.921486. PMID: 36118571 PMC9479202

[B25] Schafrum Macedo A. Cezaretti Feitosa C. Yoiti Kitamura Kawamoto F. Vinicius Tertuliano Marinho P. dos Santos Dal‐Bó Í. Fiuza Monteiro B. . (2019). Animal modeling in bone research—Should we follow the White Rabbit? Anim. Models Exp. Med. 2, 162–168. doi: 10.1002/ame2.12083. PMID: 31773091 PMC6762042

[B2] Scribd The Principles of Humane Experimental Technique | PDF | Pain | Nature. Available online at: https://www.scribd.com/document/402414979/The-Principles-of-Humane-Experimental-Technique (Accessed February 12, 2026).

[B87] Seidi A. Ramalingam M. Elloumi-Hannachi I. Ostrovidov S. Khademhosseini A. (2011). Gradient biomaterials for soft-to-hard interface tissue engineering. Acta Biomater. 7, 1441–1451. doi: 10.1016/j.actbio.2011.01.011. PMID: 21232635

[B240] Seims K. B. Hunt N. K. Chow L. W. (2021). Strategies to control or mimic growth factor activity for bone, cartilage, and osteochondral tissue engineering. Bioconjug Chem. 32, 861–878. doi: 10.1021/acs.bioconjchem.1c00090. PMID: 33856777

[B153] Shahin M. H. Liu Q. (2025). Artificial intelligence in clinical and translational science: From bench insights to bedside impact. Clin. Transl. Sci. 18, e70383. doi: 10.1111/cts.70383. PMID: 41178829 PMC12580936

[B51] Shaul J. L. Hill R. S. Bouxsein M. L. Burr D. B. Tilton A. K. Howe J. G. (2022). AGN1 implant material to treat bone loss: Resorbable implant forms normal bone with and without alendronate in a canine critical size humeral defect model. Bone 154, 116246. doi: 10.1016/j.bone.2021.116246. PMID: 34744020

[B162] Shaw R. Festing M. F. W. Peers I. Furlong L. (2002). Use of factorial designs to optimize animal experiments and reduce animal use. ILAR J. 43, 223–232. doi: 10.1093/ilar.43.4.223. PMID: 12391398

[B188] Shen H. Gardner A. M. Vyas J. Ishida R. Tawfik V. L. (2021). Modeling complex orthopedic trauma in rodents: Bone, muscle and nerve injury and healing. Front. Pharmacol. 11. doi: 10.3389/fphar.2020.620485. PMID: 33597884 PMC7882733

[B164] Shim J. Iwaya C. Ambrose C. G. Suzuki A. Iwata J. (2022). Micro-computed tomography assessment of bone structure in aging mice. Sci. Rep. 12, 8117. doi: 10.1038/s41598-022-11965-4. PMID: 35581227 PMC9114112

[B191] Sillmann Y. M. Eber P. Orbeta E. Wilde F. Gross A. J. Guastaldi F. P. S. (2025). Milestones in mandibular bone tissue engineering: A systematic review of large animal models and critical-sized defects. J. Clin. Med. 14, 2717. doi: 10.3390/jcm14082717. PMID: 40283548 PMC12027812

[B15] Stein M. Elefteriou F. Busse B. Fiedler I. A. Kwon R. Y. Farrell E. . (2023). Why animal experiments are still indispensable in bone research: A statement by the European Calcified Tissue Society. J. Bone Miner. Res. 38, 1045–1061. doi: 10.1002/jbmr.4868. PMID: 37314012 PMC10962000

[B93] Sun X. Jiao X. Yang X. Ma J. Wang T. Jin W. . (2022). 3D bioprinting of osteon-mimetic scaffolds with hierarchical microchannels for vascularized bone tissue regeneration. Biofabrication 14, 035008. doi: 10.1088/1758-5090/ac6700. PMID: 35417902

[B50] Takeuchi S. Fukuba S. Okada M. Nohara K. Sato R. Yamaki D. . (2023). Preclinical evaluation of the effect of periodontal regeneration by carbonate apatite in a canine one-wall intrabony defect model. Regener. Ther. 22, 128–135. doi: 10.1016/j.reth.2023.01.002. PMID: 36760990 PMC9898576

[B22] Tanaka S. Matsumoto T. (2021). Sclerostin: from bench to bedside. J. Bone Miner. Metab. 39, 332–340. doi: 10.1007/s00774-020-01176-0. PMID: 33206222

[B7] Tannenbaum J. Bennett B. T. (2015). Russell and Burch’s 3Rs then and now: The need for clarity in definition and purpose. J. Am. Assoc. Lab. Anim. Sci. JAALAS 54, 120–132. Available online at: https://pmc.ncbi.nlm.nih.gov/articles/PMC4382615/. 25836957 PMC4382615

[B73] Tay C. Y. Irvine S. A. Boey F. Y. C. Tan L. P. Venkatraman S. (2011). Micro-/nano-engineered cellular responses for soft tissue engineering and biomedical applications. Small 7, 1361–1378. doi: 10.1002/smll.201100046. PMID: 21538867

[B175] Teng Y. Pan D. Zhao W. (2023). Application of deep learning ultrasound imaging in monitoring bone healing after fracture surgery. J. Radiat. Res. Appl. Sci. 16, 100493. doi: 10.1016/j.jrras.2022.100493. PMID: 38826717

[B218] The ARRIVE guidelines 2.0 | ARRIVE Guidelines. Available online at: https://arriveguidelines.org/arrive-guidelines (Accessed January 26, 2026).

[B55] Unnikrishnan P. S. Iyer S. Manju V. Reshmi C. R. Menon D. Nair S. V. . (2022). Nanocomposite fibrous scaffold mediated mandible reconstruction and dental rehabilitation: An experimental study in pig model. Biomater. Adv. 133, 112631. doi: 10.1016/j.msec.2021.112631. PMID: 35527156

[B17] US - Research . Roadmap to Reducing Animal Testing in Preclinical Safety Studies ( Animal Legal & Historical Center). Available online at: https://www.animallaw.info/administrative/us-research-roadmap-reducing-animal-testing-preclinical-safety-studies (Accessed January 21, 2026).

[B26] van Agtmaal J. L. van Hoogstraten S. W. G. Arts J. J. C. (2024). Prosthetic joint infection research models in NZW rabbits: Opportunities for standardization—A systematic review. J. Funct. Biomater. 15, 307. doi: 10.3390/jfb15100307. PMID: 39452605 PMC11508679

[B57] Varut R. M. Trasca D. M. Stoica G. A. Sirbulet C. Arsenie C. C. Popescu C. (2025). Animal models as foundational tools in preclinical orthopedic implant research. Biomedicines 13, 2468. doi: 10.3390/biomedicines13102468. PMID: 41153751 PMC12561796

[B133] Vemparala B. Ji M. Mageswaran P. Knapik G. G. Dibs K. Blakaj D. M. . (2025). A computational multiscale framework for bone remodeling: coupling apparent density evolution and microscale shape optimization. Int. J. Numer. Methods Biomed. Eng. 41, e70097. doi: 10.1002/cnm.70097. PMID: 41110832 PMC12535801

[B37] Verma N. Le T. Mudge J. Nicksic P. J. Xistris L. Kasole M. . (2022). Efficacy of bone stimulators in large-animal models and humans may be limited by weak electric fields reaching fracture. Sci. Rep. 12, 21798. doi: 10.1038/s41598-022-26215-w. PMID: 36526728 PMC9758190

[B86] Vesvoranan O. Anup A. Hixon K. R. (2022). Current concepts and methods in tissue interface scaffold fabrication. Biomimetics 7. doi: 10.3390/biomimetics7040151. PMID: 36278708 PMC9624329

[B207] von der Beck B. Wissmann A. Tolba R. H. Dammann P. Hilken G. (2024). What can laboratory animal facility managers do to improve the welfare of laboratory animals and laboratory animal facility staff? A German perspective. Anim. Open Access J. MDPI 14, 1136. doi: 10.3390/ani14071136. PMID: 38612375 PMC11010866

[B170] Wang L. Lee D. J. Han H. Zhao L. Tsukamoto H. Kim Y.-I. . (2021). Application of bioluminescence resonance energy transfer-based cell tracking approach in bone tissue engineering. J. Tissue Eng. 12, 2041731421995465. doi: 10.1177/2041731421995465. PMID: 33643604 PMC7894599

[B58] Wang W. Lu J. Song Y. Zeng C. Wang Y. Yang C. . (2022). Repair of bone defects in rhesus monkeys with α1,3-galactosyltransferase-knockout pig cancellous bone. Front. Bioeng. Biotechnol. 10. doi: 10.3389/fbioe.2022.990769. PMID: 36172016 PMC9510634

[B110] Watson E. Mikos A. (2023). Advances in *In Vitro* and *In Vivo* bioreactor-based bone generation for craniofacial tissue engineering. BME Front. 4, 4. doi: 10.34133/bmef.0004. PMID: 37849672 PMC10521661

[B183] Wehrle E. Paul G. R. Tourolle né Betts D. C. Kuhn G. A. Müller R. (2021). Individualized cyclic mechanical loading improves callus properties during the remodelling phase of fracture healing in mice as assessed from time-lapsed *in vivo* imaging. Sci. Rep. 11, 23037. doi: 10.1038/s41598-021-02368-y. PMID: 34845246 PMC8630002

[B215] Wei J. Chen X. Xu Y. Shi L. Zhang M. Nie M. . (2024). Significance and considerations of establishing standardized critical values for critical size defects in animal models of bone tissue regeneration. Heliyon 10. doi: 10.1016/j.heliyon.2024.e33768. PMID: 39071581 PMC11283167

[B216] Wilson E. Ramage F. J. Wever K. E. Sena E. S. Macleod M. R. Currie G. L. (2023). Designing, conducting, and reporting reproducible animal experiments. J. Endocrinol. 258. doi: 10.1530/JOE-22-0330. PMID: 37074416 PMC10304908

[B23] Winkler D. G. Sutherland M. K. Geoghegan J. C. Yu C. Hayes T. Skonier J. E. . (2003). Osteocyte control of bone formation via sclerostin, a novel BMP antagonist. EMBO J. 22, 6267–6276. doi: 10.1093/emboj/cdg599. PMID: 14633986 PMC291840

[B202] Wolter A. Bucher C. H. Kurmies S. Schreiner V. Konietschke F. Hohlbaum K. . (2022). A buprenorphine depot formulation provides effective sustained post-surgical analgesia for 72h in mouse femoral fracture models. bioRxiv, 2022.07.05.498859. doi: 10.1101/2022.07.05.498859. PMID: . Preprint. 36882427 PMC9992384

[B67] Wragg N. M. Mosqueira D. Blokpeol-Ferreras L. Capel A. Player D. J. Martin N. R. W. . (2020). Development of a 3D tissue-engineered skeletal muscle and bone co-culture system. Biotechnol. J. 15, e1900106. doi: 10.1002/biot.201900106. PMID: 31468704

[B101] Wu Y. Ding X. Wang Y. Ouyang D. (2024). Harnessing the power of machine learning into tissue engineering: current progress and future prospects. Burns Trauma 12, tkae053. doi: 10.1093/burnst/tkae053. PMID: 39659561 PMC11630859

[B227] Wu L. Zhao J. Huang J. Huang P. Zhao H. (2025). Advances and challenges in 3D bioprinting of bone organoids: materials, techniques, and functionalization strategies. Int. J. Bioprinting, 025190183. doi: 10.36922/IJB025190183

[B48] Xu L. Zhang W. Lv K. Yu W. Jiang X. Zhang F. (2016). Peri-implant bone regeneration using rhPDGF-BB, BMSCs, and β-TCP in a canine model. Clin. Implant Dent. Relat. Res. 18, 241–252. doi: 10.1111/cid.12259. PMID: 25644231

[B65] Yamada S. Ockermann P. N. Schwarz T. Mustafa K. Hansmann J. (2023). Translation of biophysical environment in bone into dynamic cell culture under flow for bone tissue engineering. Comput. Struct. Biotechnol. J. 21, 4395–4407. doi: 10.1016/j.csbj.2023.08.008. PMID: 37711188 PMC10498129

[B83] Yang Z. Wang B. Liu W. Li X. Liang K. Fan Z. . (2023). In situ self-assembled organoid for osteochondral tissue regeneration with dual functional units. Bioact. Mater. 27, 200–215. doi: 10.1016/j.bioactmat.2023.04.002. PMID: 37096194 PMC10121637

[B99] Yang Y. Zhao Z. Qi X. Hu Y. Li B. Zhang L. (2024). Computational modeling of bone fracture healing under different initial conditions and mechanical load. IEEE Trans. Biomed. Eng. 71, 2105–2118. doi: 10.1109/TBME.2024.3361893. PMID: 38315600

[B156] Yao K. Gao P. Ni X. An J. Huang K. Helaehil J. V. . (2026). A deep learning enabled in-silico animal study approach for bone tissue engineering. Mater. Today Adv. 30, 100681. doi: 10.1016/j.mtadv.2025.100681. PMID: 38826717

[B84] Yin Y. Zhou W. Zhu J. Chen Z. Jiang L. Zhuang X. . (2025). Generation of self-organized neuromusculoskeletal tri-tissue organoids from human pluripotent stem cells. Cell Stem Cell 32, 157–171.e8. doi: 10.1016/j.stem.2024.11.005. PMID: 39657678

[B45] Yoshino Y. Miyaji H. Nishida E. Kanemoto Y. Hamamoto A. Kato A. . (2023). Periodontal tissue regeneration by recombinant human collagen peptide granules applied with β-tricalcium phosphate fine particles. J. Oral. Biosci. 65, 62–71. doi: 10.1016/j.job.2023.01.002. PMID: 36669699

[B64] Yuste I. Luciano F. C. González-Burgos E. Lalatsa A. Serrano D. R. (2021). Mimicking bone microenvironment: 2D and 3D *in vitro* models of human osteoblasts. Pharmacol. Res. 169, 105626. doi: 10.1016/j.phrs.2021.105626. PMID: 33892092

[B24] Zedda M. Babosova R. (2021). Does the osteon morphology depend on the body mass? A scaling study on macroscopic and histomorphometric differences between cow (Bos taurus) and sheep (Ovis aries). Zoomorphology 140, 169–181. doi: 10.1007/s00435-021-00516-6. PMID: 30311153

[B78] Zhang Y. (2022). Delivering Mechanical Stimulation to Cells: State of the Art in Materials and Devices Design ( Advanced Materials - Wiley Online Library). doi: 10.1002/adma.202110267 35385176

[B92] Zhang M. Lin R. Wang X. Xue J. Deng C. Feng C. . (2020). 3D printing of Haversian bone–mimicking scaffolds for multicellular delivery in bone regeneration. Sci. Adv. 6, eaaz6725. doi: 10.1126/sciadv.aaz6725. PMID: 32219170 PMC7083611

[B81] Zhang Y. Zhao Y. Sun Z. An C. Zheng G. Shen Y. . (2025). Bone-on-a-chip recapitulates dynamic bone remodeling. Acta Biomater. 208, 280–292. doi: 10.1016/j.actbio.2025.10.039. PMID: 41138824

[B230] Zhao D. Saiding Q. Li Y. Tang Y. Cui W. (2024). Bone organoids: recent advances and future challenges. Adv. Healthc Mater. 13, 2302088. doi: 10.1002/adhm.202302088. PMID: 38079529

[B31] Zhou L. Ho K.-W. K. Zheng L. Xu J. Chen Z. Ye X. . (2024). A rabbit osteochondral defect (OCD) model for evaluation of tissue engineered implants on their biosafety and efficacy in osteochondral repair. Front. Bioeng. Biotechnol. 12. doi: 10.3389/fbioe.2024.1352023. PMID: 38766649 PMC11099227

[B1012] Zhou Y. Ping X. Guo Y. Heng B. C. Wang Y. Meng Y. . (2023). Assessing Biomaterial-Induced Stem Cell Lineage Fate by Machine Learning-Based Artificial Intelligence. Advanced Materials 35, (19), e2210637. doi: 10.1002/adma.202210637 36756993

